# Essential role of CK2α for the interaction and stability of replication fork factors during DNA synthesis and activation of the S-phase checkpoint

**DOI:** 10.1007/s00018-022-04374-3

**Published:** 2022-06-04

**Authors:** Barbara Guerra, Thomas K. Doktor, Sabrina B. Frederiksen, Kumar Somyajit, Brage S. Andresen

**Affiliations:** Department of Biochemistry and Molecular Biology, University of Southern Denmark, Odense, Denmark

**Keywords:** CK2α, S-phase checkpoint, CDC45, CLSPN, MCM7

## Abstract

The ataxia telangiectasia mutated and Rad3-related (ATR)-CHK1 pathway is the major signalling cascade activated in response to DNA replication stress. This pathway is associated with the core of the DNA replication machinery comprising CDC45, the replicative MCM2-7 hexamer, GINS (altogether forming the CMG complex), primase–polymerase (POLε, -α, and -δ) complex, and additional fork protection factors such as AND-1, CLASPIN (CLSPN), and TIMELESS/TIPIN. In this study, we report that functional protein kinase CK2α is critical for preserving replisome integrity and for mounting S-phase checkpoint signalling. We find that CDC45, CLSPN and MCM7 are novel CK2α interacting partners and these interactions are particularly important for maintenance of stable MCM7–CDC45, ATRIP–ATR–MCM7, and ATR–CLSPN protein complexes. Consistently, cells depleted of CK2α and treated with hydroxyurea display compromised replisome integrity, reduced chromatin binding of checkpoint mediator CLSPN, attenuated ATR-mediated S-phase checkpoint and delayed recovery of stalled forks. In further support of this, differential gene expression analysis by RNA-sequencing revealed that down-regulation of CK2α accompanies global shutdown of genes that are implicated in the S-phase checkpoint. These findings add to our understanding of the molecular mechanisms involved in DNA replication by showing that the protein kinase CK2α is essential for maintaining the stability of the replisome machinery and for optimizing ATR-CHK1 signalling activation upon replication stress.

**Supplementary Information:**

The online version contains supplementary material available at 10.1007/s00018-022-04374-3.

## Introduction

DNA replication occurs in the S-phase; however, the assembly of pre-replicative complexes (pre-RCs) begins several hours earlier in the cells in preparation to this event. The multi-subunit origin recognition complex (ORC) bound to chromatin primes this process by facilitating the recruitment of Cdc6, Cdt1 and the minichromosome maintenance (MCM2-7) proteins to distinct replication origin sites on the DNA. It follows recruitment of additional factors including cell division cycle 45 (CDC45) and the go-ichi-ni-san (GINS) complex to form the so-called CMG complex that represents the core of helicase activity [1, 2]. Several lines of evidence indicated that MCM2-7 are replicative helicases, which in combination with the aforementioned essential replisome factors (i.e. CDC45 and GINS) unwind segments of the DNA double helix during the replication process [1, 2]. Initiation of DNA replication during S-phase also requires the phosphorylation of components of the pre-RC catalysed by cyclin E-CDK2 and Cdc7-Dbf4 and subsequent association of three DNA polymerases (i.e. Pol α, Pol ε and Pol δ) at origin of replication. At this point replication can be started [3, 4].

During DNA synthesis, the unwinding of DNA leaves a single strand vulnerable. Eukaryotic cells are equipped with surveillance mechanisms coupled to the replisome that detect errors or problems occurring during this process and arrest the cell cycle allowing time for DNA repair to ensure the stability of the genome. In the eventuality of DNA damage and, specifically, DNA replication stress, long stretches of single-stranded DNA (ssDNA) coated by replisome protein A (RPA) are formed and a checkpoint signalling cascade is activated.

The ataxia telangiectasia mutated and Rad3-related kinase (ATR) and its obligate binding partner ATRIP, are recruited to RPA-coated ssDNA stretches and this is followed by binding of 9–1–1 (Rad9–Hus1–Rad1) heterotrimeric clamp and its clamp-loader Rad17 to sites of stalled replication fork [5, 6]. The presence of 9–1–1 on chromatin facilitates the interaction of ATR–ATRIP and a group of checkpoint mediators including TopBP1 and Mrc1/CLSPN [4].Compelling evidence has indicated that CLSPN is associated with chromatin and interacts with the checkpoint kinase CHK1 in response to stalled replication fork promoting checkpoint signalling at the replisome [7–10]. Here, one important role of ATR in response to replication block is the phosphorylation of several replication proteins including CLSPN, MCM2-7, H2AX and CHK1 [11]. Phosphorylation of the effector kinase CHK1 at S345 and S317 by ATR enhances its kinase activity, resulting in dissociation from chromatin, phosphorylation of CDC25A-C phosphatases, which prevent the progression of the cell cycle [11–13]. Efficient phosphorylation of CHK1 in response to replication block also requires the intervention of other factors, notably, MCM7, Rad17, TopBP1, the MRN complex and CLSPN. Similar to yeast, vertebrate ATR primarily utilizes adaptor CLSPN to mediate the activation of CHK1 by tethering it in proximity to ATR allowing for extensive phosphorylation and full activation of the effector kinase [14, 15].

CK2 is an evolutionarily conserved serine-threonine protein kinase that has been linked to the regulation of various intracellular processes including DNA transcription, cell cycle transition, protein translation, cell survival and cell death [16–20]. CK2 has been traditionally described as a constitutively active heterotetrametric enzyme composed of two catalytic subunits α and/or α’ and two regulatory β-subunits. However, accumulating evidence has indicated that these proteins may exist as individual isoforms in cells [21, 22]. Data based on gene targeting by homologous recombination has strengthen the notion that the three CK2 subunits might be also functionally specialized in vivo. For instance, although the *CK2α* and *CK2α’* genes share approximately 90% sequence identity, genetic studies have shown that the phenotypic response to gene disruption is markedly different. Mice lacking *CK2α* die in mid gestation and show abnormality in a number of tissues and organs including the neural tube and heart [23] whereas homozygous deletion of *CK2α'* results in viable offspring although the male mice are infertile and affected by globozoospermia [24]. CK2 has been shown to positively regulate cell cycle progression in cancer cells by interacting and/or phosphorylating a number of proteins including p53, PLK1, CHK1, Wee1, MDC1, DNA-PK, and 53BP1 [25–33], but the function of CK2 in non-cancerous cells has not been explored to the same extent.

We previously showed that down-regulation of CK2α in normal myoblast cells derived from rat heart tissue (hereafter referred to as H9c2-CK2α-44) results in decreased cell proliferation, delayed G1/S-phase transition and defective response to mild DNA replication stress [22], which if unaddressed, could have serious implications for the genome stability of mammalian cells. Decreased proliferation of cardiomyocytes was also shown in vivo with CK2α-knockout mice strongly supporting the notion that this process is regulated by this protein kinase [22].

The aim of the present study was to shed light on the underlying molecular mechanisms by which CK2α regulates the DNA replication machinery and replication checkpoint activation. Our results show an interplay between CK2α and the replisome and that this protein kinase plays a critical role in the activation of the ATR-CHK1-mediated signalling pathway by ensuring stable association of replication factors on chromatin in response to a stalled DNA replication fork.

## Materials and methods

### Cell culture and treatments

The cell line H9c-2 originated from rat heart was purchased from the American Type Culture Collection (ATCC, Rockville, MD, USA) and used for the generation of the cell line H9c2-CK2α-44 employed in this study [22, 34]. The cell line was cultivated at 37 ºC under a 5% CO_2_ atmosphere in Dulbecco’s modified Eagle’s medium (DMEM, Invitrogen, Taastrup, Denmark) supplemented with 10% foetal bovine serum (FBS, Biochrom AG, Berlin, Germany). Cells were added 1 μg/ml doxycycline (Dox, Sigma-Aldrich, Brøndby, Denmark) to induce down-regulation of CK2α expression as previously described [22]. The human osteosarcoma U2OS cell line was purchased from ATCC and cultivated in DMEM supplemented with 10% foetal bovine serum at 37ºC under a 5% CO_2_. Where indicated, cells were transfected for 72 h with a set of four small interfering RNA duplexes (ON-TARGET plus SMART pools, Dharmacon—Horizon Discovery, Lafayette, CO, USA) directed against human *CK2α* mRNA or rat *ATR* mRNA using Lipofectamine RNAiMAX transfection reagent (Thermo Scientific, Waltham, MA, USA). Control experiments were performed employing cells transfected with scramble-siRNA (Dharmacon–Horizon Discovery). The expression of FLAG-CLSPN and HA-CHK1 was obtained by transfecting cells with plasmids coding for FLAG-CLSPN [Addgene plasmid # 12659, [35]] and HA-CHK1 [27], respectively, in the presence of Lipofectamine 3000 transfection reagent (Thermo Scientific) for 48 h following the manufacturer’s recommendations.

Cell synchronization in G0/G1-phase was obtained by growing cells in the presence of 0.1% FBS for 48 h before adding complete growth medium. Cells were subsequently harvested at different time points as indicated in the figures. Hydroxyurea (HU), nocodazole (Noc) and aphidicolin (Aph) were purchased from Sigma-Aldrich. Exposure to 60 J/m^2^ ultraviolet (UV) irradiation was carried out with the Stratalinker UV Crosslinker (Stratagene, CA, USA).

### Cell cycle analysis

Cell cycle analysis was performed with a FACSCalibur flow cytometer (BD Biosciences, Franklin Lake, NJ, USA) after staining of the cells with propidium iodide as previously described [31]. Acquired data were processed by Cell Quest Pro Analysis software (BD Biosciences). For each measurement, 10,000 events were analysed. Values indicated in the graphs are expressed in percentage of total number of cells. Determination of the percentage of cells with reduced/fragmented DNA content (i.e., sub-G1) indicates cells undergoing cell death.

### BrdU assay

The assay was carried out using the 5-bromo-2ʹ-deoxyuridine (BrdU) Cell Proliferation Assay kit (6813, Cell Signaling Technology, MA, USA) following the manufacturer’s instructions. In brief, cells were incubated with 3 mM HU for 12 h. They were, then, washed with warm PBS and, subsequently, incubated with growth medium containing BrdU at 37 °C for additional 10 h. After fixation and denaturation, cells were incubated with anti-BrdU for 1 h at room temperature and, subsequently, with anti-mouse IgG, HRP-conjugated for 30 min at room temperature. Conjugates were visualized by adding TMB substrate. Colorimetric reactions were quantified by measuring the absorbance at 450 nm. Background signal-intensity where the incubation with the primary antibody was omitted was subtracted from all measurements.

### IncuCyte S3 image capture and analysis

Cells were seeded in 96-well plates in the presence or absence of Dox. 48 h after seeding, cells were added HU or vehicle and imaged using the phase contrast channel in the IncuCyte S3 platform (Sartorius, Göttingen, Germany). Four phase contrast images/well from distinct regions were taken at regular intervals using a 10 × magnification objective. Images were analysed employing the IncuCyte S3 image analysis software and Microsoft Excel software as previously described [34].

### Preparation of whole cell lysate and nuclear extract, Western blot analysis, immunoprecipitation, kinase assay and antibodies

Harvested cells were processed for SDS-PAGE and Western blot analysis essentially as previously described [28]. Proteins were detected by incubating PVDF membranes with the following primary antibodies: rabbit polyclonal anti-phospho-NF-κB S529 (ab47395, Abcam, Cambridge, United Kingdom), rabbit monoclonal anti-phospho-CHK1 S345 (2348, Cell Signaling Technology), mouse monoclonal anti-CHK1 (2360, Cell Signaling Technology), rabbit monoclonal anti-cyclin E1 (20808, Cell Signaling Technology), mouse monoclonal anti-p53 (2524, Cell Signaling Technology), rabbit polyclonal anti-phospho-p53 S15 (9284, Cell Signaling Technology), rabbit polyclonal anti-Cdc7 (3603, Cell Signaling Technology), rabbit monoclonal anti-NF-κB (8242, Cell Signaling Technology), rabbit monoclonal anti-CDC45 (11881, Cell Signaling Technology), rabbit monoclonal anti-phospho-histone H2A.X S139 (9718, Cell Signaling Technology), mouse monoclonal anti-α-Tubulin (3873, Cell Signaling Technology), rabbit monoclonal anti-histone H3 (4499, Cell Signaling Technology); rabbit polyclonal anti-cyclin A (sc-751, Santa Cruz Biotechnology, Heidelberg, Germany), mouse monoclonal anti-ATR (sc-515173, Santa Cruz Biotechnology), mouse monoclonal anti-ATRIP (sc-365383, Santa Cruz Biotechnology), mouse monoclonal anti-MCM7 (sc-9966, Santa Cruz Biotechnology), mouse monoclonal anti-CLSPN (sc-376773, Santa Cruz Biotechnology), goat polyclonal anti-MCM3, mouse monoclonal anti-CDK2 (sc-6248, Santa Cruz Biotechnology), mouse monoclonal anti-HDAC2 (sc-9959, Santa Cruz Biotechnology), and mouse monoclonal anti-β-actin (A-5441, Sigma-Aldrich). Rabbit polyclonal anti-CK2α, rabbit polyclonal anti-CK2α’ and mouse monoclonal anti-CK2β were obtained as previously described [22].

Nuclear extracts were prepared essentially as described in [36]. Briefly, cells were resuspended in buffer A [10 mM Hepes, pH 7.9, 10 mM KCl_2_, 1.5 mM MgCl_2_, 0.34 M sucrose, 10% glycerol, 1 mM DTT, proteases inhibitor cocktail (Roche, Basel, Switzerland), 100 nM okadaic acid (Sigma-Aldrich)]. After adding 0.1% Triton X-100, cells were incubated for 5 min on ice. Nuclei (P1) were separated from the soluble fraction (S1) by centrifugation (1,300 × g, 5 min, 4 °C). The soluble protein fraction was subjected to centrifugation at 20,000×*g* (15 min, 4 °C). This resulted in the clarified soluble protein fraction (S2). The P1 nuclear fraction was resuspended in buffer A (washing step), and then lysed in the presence of buffer B (3 mM EDTA, 0.2 mM EGTA, 1 mM DTT, proteases inhibitor cocktail, 100 nM okadaic acid) on ice for 30 min. The soluble (S3) and insoluble nuclear fractions (P2) were separated by centrifugation (1400×*g*, 5 min, 4 °C). Pellet P2 was washed once with buffer B and centrifuged at 1700×*g*, 5 min, 4 °C. The resulting pellet P3 (chromatin-enriched fraction) was resuspended in sample buffer and briefly sonicated before loading into SDS–polyacrylamide gel.

Immunoprecipitation experiments were performed essentially as previously described [37] employing 1 mg whole cell lysate and rabbit monoclonal anti-CDC45 antibody (11881, Cell Signaling Technology), rabbit monoclonal anti-HA antibody (3724, Cell Signaling Technology), rabbit polyclonal anti-ATRIP antibody (A7139, ABclonal Technology, MA, USA), mouse monoclonal anti-CLSPN antibody (sc-376773, Santa Cruz Biotechnology), goat polyclonal anti-CK2α antibody (sc-6479, Santa Cruz Biotechnology) or rabbit polyclonal anti-CK2α antibody as indicated in the figure legends. Protein A-Agarose and protein G-Agarose were purchased from Roche.

**Fig. 1:**
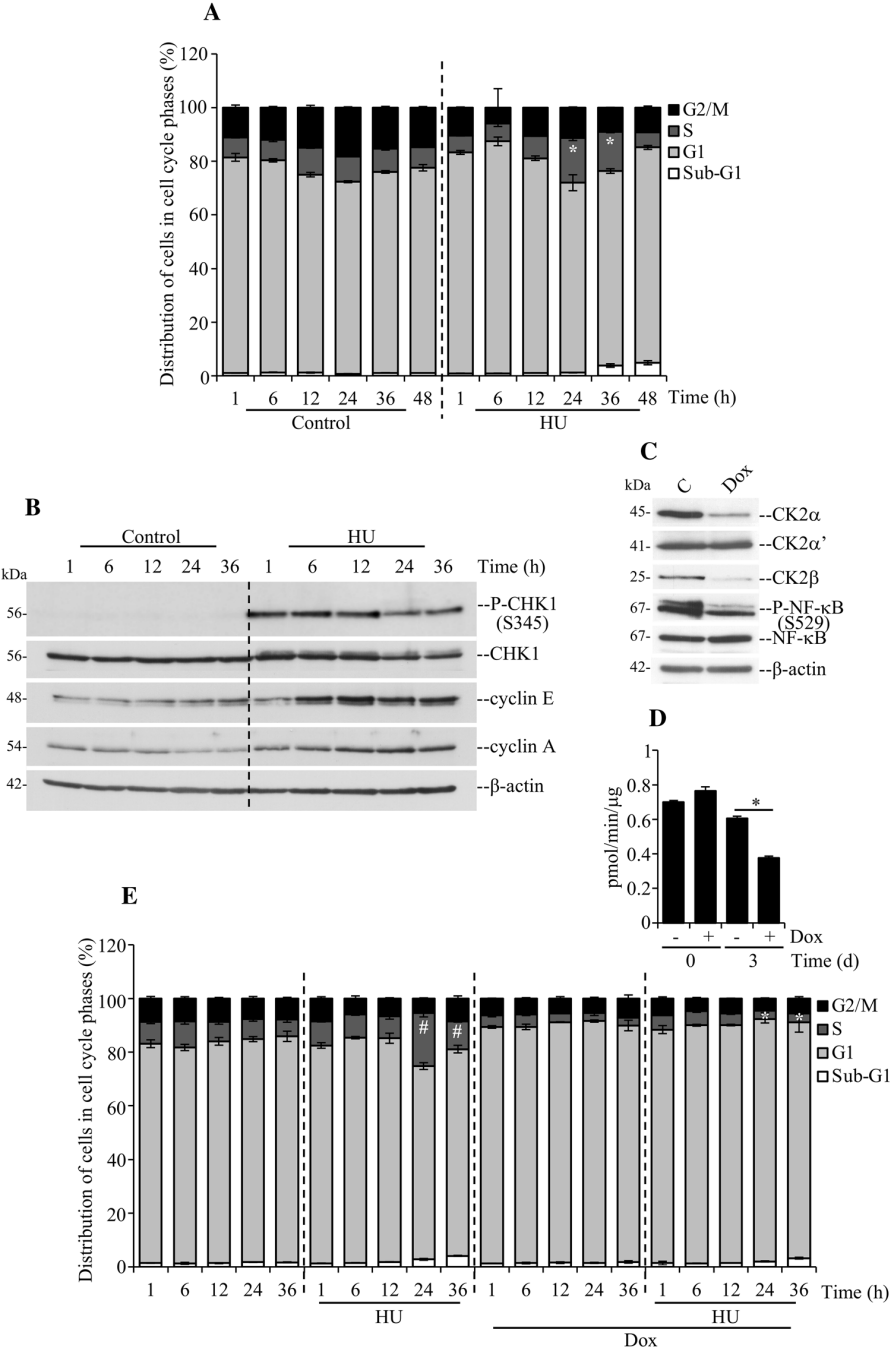
Compromised DNA damage response to replication stress induced by HU in myoblasts with down-regulation of CK2α. **A** Flow cytometry analysis of H9c2-CK2α-44 cells incubated with vehicle or 3 mM hydroxyurea (HU) for the indicated times. Cell cycle analysis was performed following propidium iodide staining. Distribution of cells in cell cycle phases is indicated in percentage. *P < 0.05 with respect to control cells at 24 h, and 36 h, respectively. **B** Western blot analysis of whole lysate from cells treated as indicated in **A** was carried out employing the indicated antibodies. β-actin detection served as loading control. **C**, **D** Analysis of whole lysate from cells treated with vehicle or 1 μg/ml doxycycline (Dox) for 72 h. Antibodies directed against the indicated proteins were employed in the Western blot analysis (**C**). Detection of phosphorylated NF-κB at S529 served as an indication of residual CK2 kinase activity in the cells following Dox treatment (**C**). Residual activity of the endogenous CK2 was also quantified by radioactive-based kinase assay (**D**). *P < 0.005. **E** Cells were incubated with vehicle or 1 μg/ml Dox for 48 h and then added 3 mM HU and subsequently harvested at various time points as indicated in the figure. The number of cells in the various phases of the cell cycle is expressed in percentage. ^#^*P* < 0.05 with respect to control cells at 24 h, and 36 h, respectively; *P < 0.005 with respect to cells incubated with HU for 24 h, and 36 h, respectively. All experiments were performed three times obtaining similar results. One representative experiment is shown

Kinase assay was performed essentially as described in [37] employing 10 μg whole cell lysate and 190 μM synthetic peptide RRRDDDSDDD as a substrate. Values shown in Fig. 1D are the result of three independent experiments.

### Immunostaining

Cells were grown on cover slips and subsequently incubated with rabbit polyclonal anti-phospho-histone H2A.X S139 (2577, Cell Signaling Technology) followed by incubation with biotinylated swine anti-rabbit IgG (E 0431, Dako, Glostrup, Denmark) and streptavidin-conjugated Alexa 555 (Invitrogen) as previously described [38]. Cells were counterstained with Hoechst 33258 dye (Sigma-Aldrich), analysed with a DMRBE microscope equipped with a Leica DFC420C camera (Leica, Denmark) and processed using ImageJ software (NIH, MD, USA). Quantification of positive signals was carried out by manual counting by two independent investigators.

### RNA-sequencing library preparation, sequencing, and data analysis

The followed procedure was based upon our previously published work [22]. In brief, total RNA was extracted from cells using Isol-RNA lysis reagent (AH Diagnostics, Aarhus, Denmark). The RNA concentration, purity and integrity were analysed on Nanodrop and Agilent 2100 Bioanalyzer using the RNA 6000 nano kit (Agilent Technologies, Inc., Santa Clara, CA, USA). RNA used for sample preparation had a RIN ≥ 8.0 and a 28 s/18 s ratio ≥ 1.8. Sample preparation was performed as described in the NEBNext ultra II RNA library prep kit (Illumina). The amplified libraries were validated by Agilent 2100 Bioanalyzer using a DNA 1000 kit from Agilent Technologies and quantified by qPCR using the KaPa Library Kits (KaPa Biosystems, Wilmington, MA, USA). Libraries were loaded on the flow cell 2 × 150 bp sequencing on Illumina Novaseq 6000. For data analysis, samples were first trimmed to remove NEBNext adaptor using bbduk.sh [39] and, subsequently, mapped to the rat transcriptome using salmon [40] version 1.3.0 with transcript annotation from Ensembl ver. 103. Because *CHK1* was missing in the Ensembl rat annotation, we manually curated the *CHK1* locus and found that *CHK1* had been merged with *Stt3a* in a previous build. We therefore manually assigned the two *CHK1* transcripts to the *CHK1* gene using a previous *CHK1* Ensembl ID (ENSRNOG00000008181) and have contacted Ensembl about this issue. Subsequently, gene counts were obtained by merging transcript counts using tximport [41] and differential gene expression analysed with DESeq2 [42] with shrinkage of fold-changes using apeglm [43]. Following pair-wise comparisons, we took the significantly differentially expressed genes and used them in a SOM analysis to identify gene patterns associated with specific conditions using the kohonen package [44, 45]. Briefly, mean expression values from each condition were scaled and centred around 0 to account for differences in expression between different genes. We then defined a map of 4 by 4 nodes and clustered genes to these nodes using the self-organizing map method. Following that, we clustered the nodes into 12 clusters using hierarchical clustering, to merge similar nodes. We performed pathway analyses on these 12 clusters using clusterProfiler [46] and made plots with enrichplot (https://yulab-smu.top/biomedical-knowledge-mining-book/) and pathview [47]. To visualize patterns across clusters, we selected the top-5 enriched pathways from each cluster and used a dotplot to plot overall patterns. Because some pathways were enriched in multiple clusters, the number of pathways plotted for an individual cluster is sometimes more than 5, but all pathways were in the top-5 enriched pathways in at least one cluster.

### Quantitative image-based cytometry (QIBC)

QIBC was performed as previously described [48]. Briefly, images were acquired with a ScanR inverted microscope high-content screening station (Olympus) equipped with wide-field optics, air objective, fast excitation and emission filter-wheel devices for DAPI, FITC, Cy3, and Cy5 wavelengths, an MT20 illumination system, and a digital monochrome Hamamatsu ORCA-Flash 4.0LT CCD camera. Images were acquired in an automated fashion with the ScanR acquisition software (Olympus, 3.2.1). 81 images were acquired containing at least 10,000 cells per condition. Acquisition times for the different channels were adjusted for non-saturated conditions in 12-bit dynamic range, and identical settings were applied to all the samples within one experiment. Images were processed and analysed with ScanR analysis software. First, a dynamic background correction was applied to all images. The DAPI (Sigma-Aldrich) signal was then used for the generation of an intensity-threshold-based mask to identify individual nuclei as main objects. This mask was then applied to analyse pixel intensities in different channels for each individual nucleus. These values were then exported and analysed with TIBCO Software, version 11. This software was used to quantify absolute, median, and average values in cell populations and to generate all color-coded scatter plots. Within one experiment, similar cell numbers were compared for the different conditions (at least 4.000–5.000 cells), and for visualization low x-axis jittering was applied (random displacement of objects along the x axis) to make overlapping markers visible.

### Statistical analysis

All values are expressed as mean ± standard deviation. All experiments were independently repeated at least three times. Student’s *t* test method was applied when compared two groups of data. The statistical significance of values is indicated in the figure legends. All statistical analyses were carried out using Excel software.

## Results

### CK2α is required for activation of the DNA replication stress checkpoint in myoblasts

Earlier work in our laboratory showed that inducible down-regulation of CK2α in myoblasts (i.e., H9c2-CK2α-44 cells) in the presence of doxycycline (Dox) leads to significant inhibition of cell growth and enhanced sensitivity to induction of mild DNA replication stress [22]. To determine whether CK2α is required for the activation of S-phase checkpoint, we incubated cells with hydroxyurea (HU) for variable lengths of time to induce stalled replication forks. HU inhibits ribonucleotide reductase causing depletion of deoxynucleotide triphosphates and, thereby, arrests active DNA synthesis [49]. Flow cytometry analysis of cells harvested at different time points showed accumulation in G1/S- and S-phases for up to 24 h before resuming the cell cycle suggesting induction of DNA replication block and/or slowing down of fork progression (Fig. 1A). Western blot analysis of whole lysates from cells treated as indicated above showed that HU activated a checkpoint response evidenced by increased ATR-mediated phosphorylation of CHK1 at S345 and concomitant accumulation of cyclin E and cyclin A suggesting cell cycle arrest (Fig. 1B). To examine the DNA replication checkpoint response in cells depleted of CK2α by Dox treatment (Fig. 1C and D), we measured cells’ DNA content by flow cytometry according to treatments indicated in Fig. 1E. The analysis revealed a slightly increased G1 population following treatment with Dox as compared to control cells that confirmed our previous findings [22, 34]. Conversely, cells with down-regulation of CK2α and additionally incubated with HU, exhibited marked reduced response to depletion of deoxynucleotides. Ideally, we would wish to rescue the RNAi phenotype by the expression of the target gene (*CK2α*) in a form refractory to the shRNA effect. Such an approach is presently complicated due to varying uptake of the cDNA-expressing construct in individual cells, leading to a spectrum of phenotypes within the cell population. Approaches to obtain consistently regulatable expression of both shRNA and cDNA are presently being developed so that the rescue of the RNAi effects can be reliably tested.

**Fig. 2:**
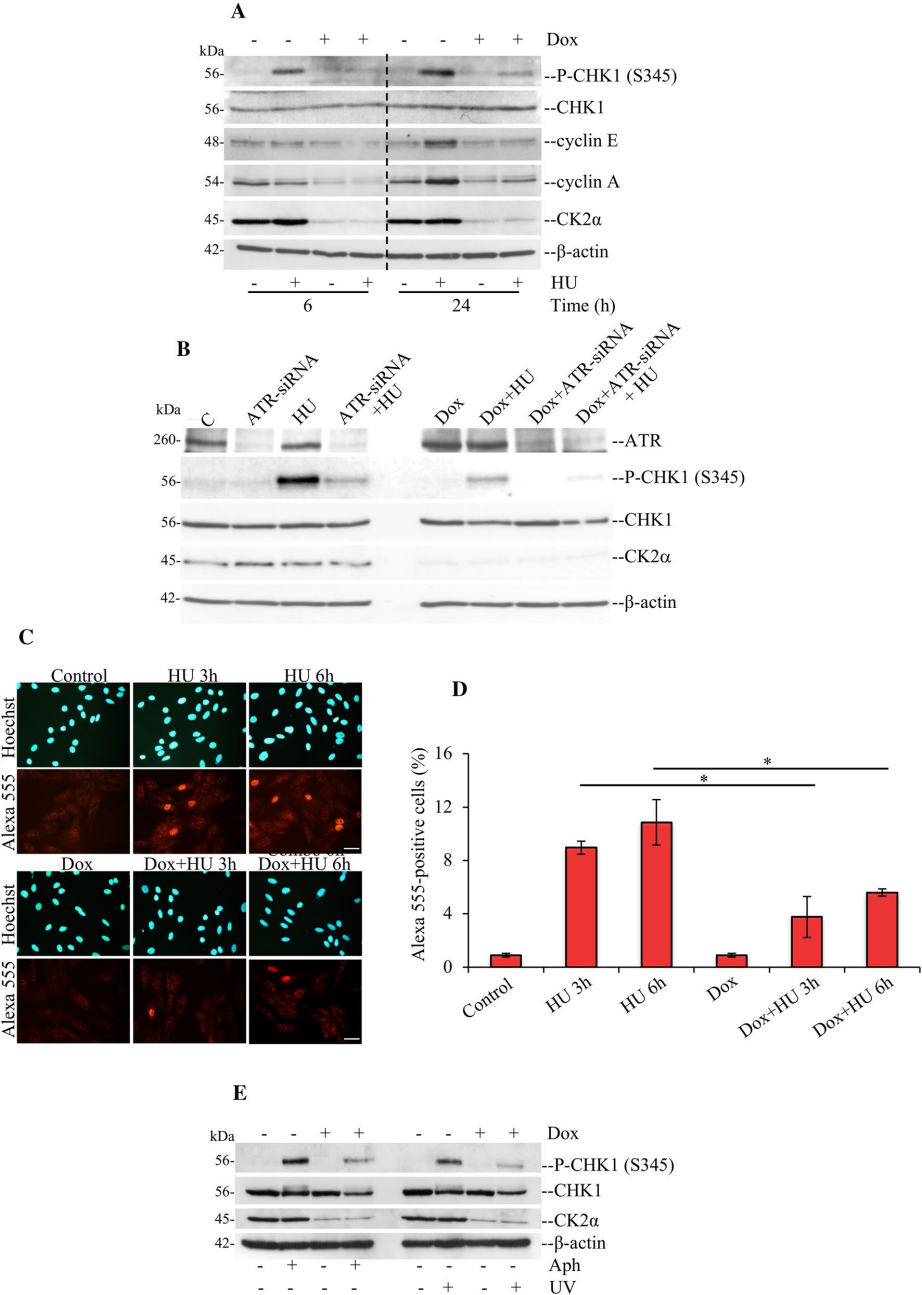
Down-regulation of CK2α correlates with defective ATR-mediated phosphorylation of CHK1 at the activating S345 following treatment with agents inducing DNA replication stress. **A** H9c2-CK2α-44 cells were treated with vehicle or 1 μg/ml Dox for 48 h and subsequently incubated with 3 mM HU for 6 h and 24 h, respectively. Whole cell lysates were analysed by Western blot employing antibodies against the indicated proteins. Detection of β-actin was used as loading control. **B** Western blot analysis of whole lysate derived from cells transfected with siRNA against ATR (ATR-siRNA), scramble siRNA (**C**), 3 mM HU for 6 h or a combination of ATR-siRNA and HU as indicated in the figure, was carried out with antibodies against the indicated proteins. **C** Immunostaining of H9c2-CK2α-44 cells with anti-CHK1 antibody binding to the phosphorylated form of the kinase at serine 345 [P-CHK1 (S345)]. Cells were treated essentially as indicated in **A** prior fixation and staining after 3 h and 6 h of incubation with 3 mM HU, respectively. Nuclei were visualized by Hoechst 33258 staining. Photos were taken at 20 × magnification. Scale bar represents 50 μm. **D** Quantification of P-CHK1 (S345)-positive cells treated as described in **A**, was performed by manual counting and expressed as percentage of the total number of cells in each sample. Bars indicate mean values ± standard deviation from three independent experiments. Asterisk denotes statistical significance: *P < 0.05. **E** Cells were treated with vehicle or 1 μg/ml Dox for 48 h and subsequently incubated with 5 μM aphidicolin (Aph) or exposed to 60 J/m^2^ UV as indicated in the figure. After 6 h, cells were harvested and processed for Western blot analysis of proteins indicated in the figure. All experiments were repeated at least three times obtaining similar results

We additionally analysed whole extracts by Western blot from cells treated as indicated in Fig. 2A. We found that while in control cells, HU induced marked CHK1 phosphorylation at S345, and upregulation of cyclin E and cyclin A at 24 h treatment, this effect was abrogated following down-regulation of CK2α. Interestingly, while depletion of ATR alone largely abrogated Chk1 phosphorylation at S345, down-regulation of CK2α and ATR together led to a further decline in CHK1 phosphorylation, suggesting that CK2α is involved in ATR-dependent activation of CHK1 in response to replication stress (Fig. 2B). Results obtained from immunofluorescence staining (Fig. 2C and D and Suppl. Fig S1A) confirmed the analysis by Western blot (Fig. 2A) and revealed that approx. 9% and 11% of cells were positively stained for CHK1 S345 at 3 h and 6 h after HU, respectively, whereas only approx. 4% and 6% of cells were positive for CHK1 S345 when CK2α was down-regulated. Overall, these results indicate that CK2α is required for optimal ATR-CHK1-mediated checkpoint activation.

DNA replication stress can be induced by agents and conditions other than HU. Hence, to verify whether the reported effect was linked to DNA replication stress and not specifically to HU treatment, we verified whether aphidicolin treatment (Aph), an inhibitor of DNA polymerases, or exposure to 60 J/m^2^ UV irradiation [49, 50] would also induce phosphorylation of CHK1 in H9c2-CK2α-44 cells. Cell exposure to Aph or UV irradiation resulted in prompt activation of ATR-CHK1 checkpoint signalling as shown by increasing phosphorylation of CHK1 at S345 (Fig. 2E and Suppl. Fig S2). However, CHK1 phosphorylation was significantly abrogated in cells with down-regulation of CK2α indicating that CK2α is required for the activation of a checkpoint response in cells undergoing DNA replication stress (Fig. 2E).

### Global gene expression profiling indicates an important role of CK2α in the regulation of genes controlling DNA replication

**Fig. 3:**
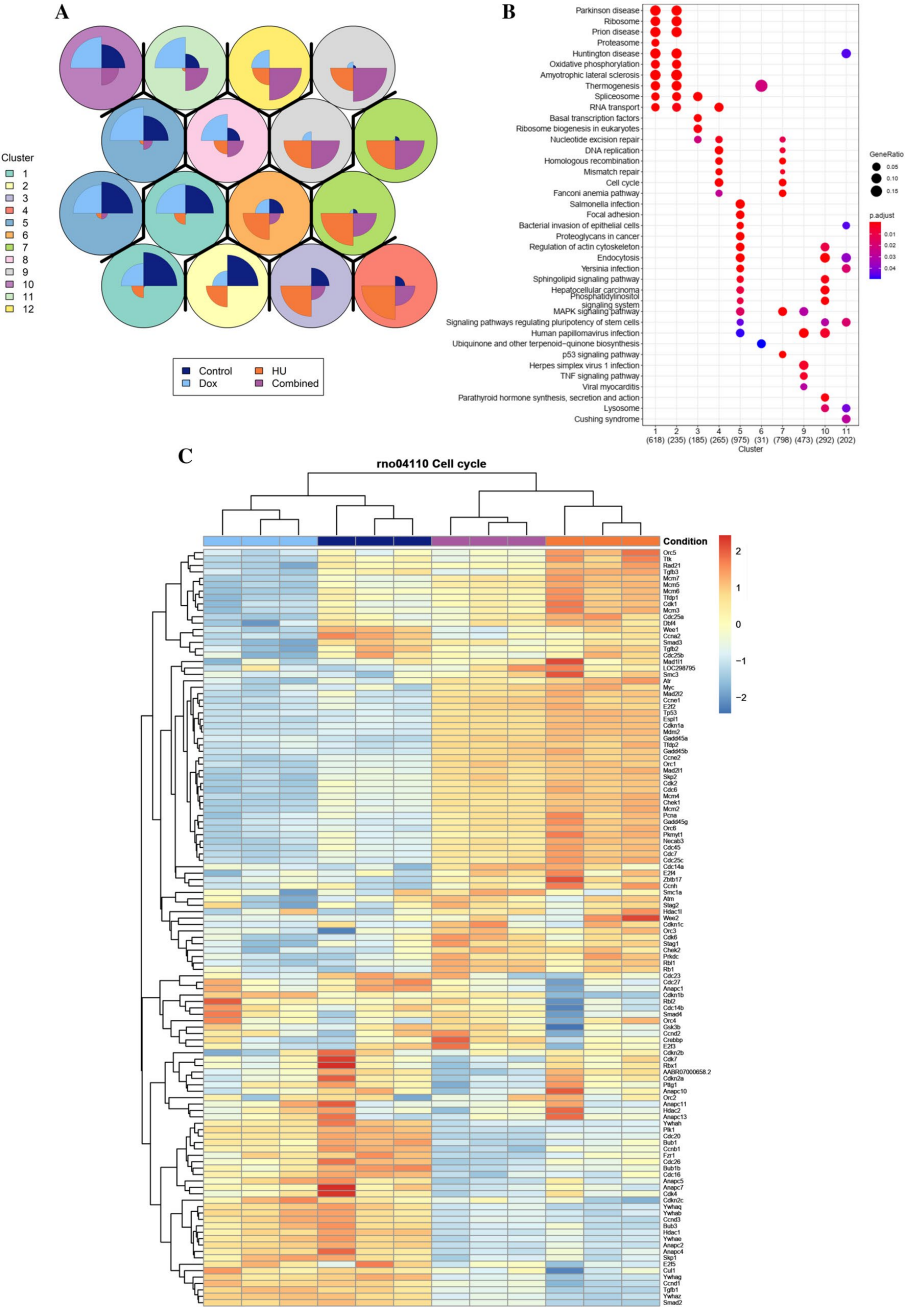
Differential gene expression analysis by RNA-sequencing reveals significant alterations in the expression of genes implicated in cell cycle regulation. Cells were treated with vehicle or 1 μg/ml Dox for 72 h and added 3 mM HU in the last 24 h of incubation time. **A** Self-organizing map analysis of genes with statistically different expression in pairwise comparisons. Hierarchical clustering was used to obtain the 12 overall clusters indicated by colouring of individual nodes as well as dark lines between each cluster. Standardized gene expression values were used as input and general expression of genes within each cluster is shown in pie-charts within each node, segmented into conditions (*n* = 3, condition indicated by colour legend at the bottom), with a pronounced slice indicating high expression and smaller slices indicating low expression, such that in the case of cluster 1, genes are expressed more in in control and Dox-treated conditions than in HU and combination treatments while in cluster 9, the opposite is true. **B** Dot plot of KEGG pathways enriched within gene clusters (adj. *P* value < 0.05), each pathway was among the five most enriched pathways in at least one cluster. **C** Heatmap of scaled log-regularized gene expression of genes in the KEGG cell cycle pathway (rno04110). The condition is indicated above using the same colour legend as in **A**

To examine CK2α’s role in the regulation of DNA replication and activation of S-phase checkpoint under DNA replication stress, we carried out RNA-sequencing on the H9c2-CK2α-44 myoblast cells [22] treated with either Dox to induce down-regulation of CK2α, HU to induce replication stress, or a combination of both agents and compared to vehicle-treated cells as control. We, then, identified gene clusters associated with each group using self-organizing maps (Fig. 3A) and identified KEGG pathways enriched within each cluster (Fig. 3B). We found that pathways associated with DNA replication and cell cycle were enriched within clusters 4 and 7, that were generally characterized by genes with higher expression in cells treated with HU as compared to controls, but less so in cells treated with both HU and Dox. More specifically, a closer examination of genes within the cell cycle pathway (Fig. 3C, Suppl. Figs S3 and S4) showed that the combination of HU and Dox led to a normalization of the expression of genes associated with DNA replication, such as the minichromosome maintenance proteins (*MCMs*) 3, 5, 6 and 7, origin recognition complex protein 5 (*Orc5),* transforming growth factor beta 3 (*Tgfb3*), double-strand-break repair protein rad21 homolog (*Rad21*), cyclin-dependent kinase 1 (*CDK1*), cell division cycle 25A (*CDC25A*), Wee1 G2 checkpoint kinase (*Wee1*), cyclin A2 (*Ccna2*), and cell division cycle 45 (*CDC45*). These were all up-regulated in HU-treated cells and down-regulated in Dox-treated cells. Conversely, we also observed normalization of expression of genes otherwise down-regulated in cells treated with HU alone, such as the cyclin-dependent kinase inhibitor 1B (*Cdkn1b*), cell division cycle 14B (*Cdc14b*), RB transcriptional corepressor like 2 (*Rbl2*) and SMAD family member 4 (*SMAD4*).

Altogether, these findings provide molecular evidence as to how the normal cellular response to DNA replication stress, which results in increased expression levels of many of the DNA replication machinery genes, is compromised in cells with perturbed CK2α levels resulting in delayed G1 to S-phase transition and defect S-phase checkpoint activation.

### CK2α is required for ATR-CHK1-mediated checkpoint activation in response to DNA replication stress

**Fig. 4:**
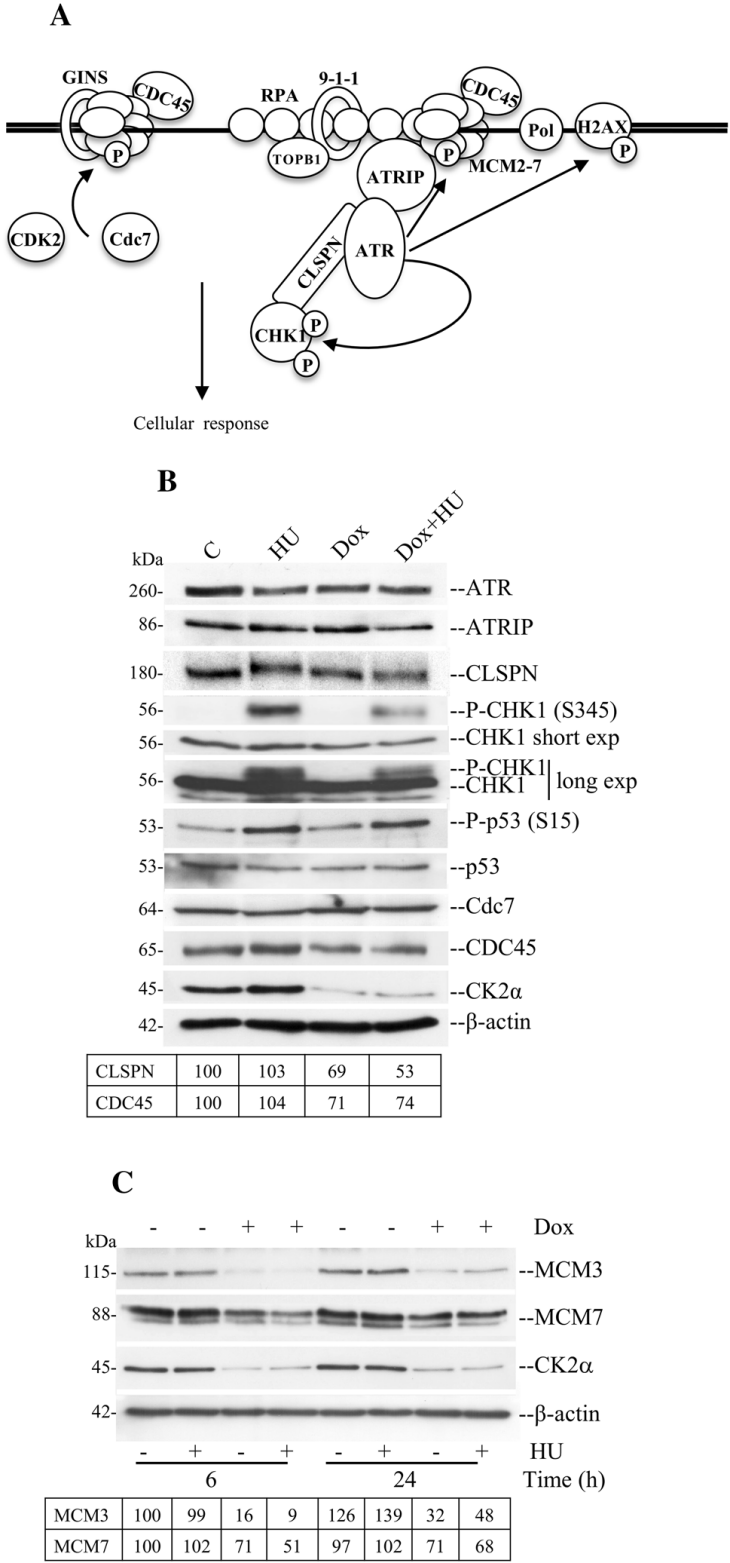
Down-regulation of CK2α affects the expression of cell cycle regulatory proteins implicated in the response to DNA replication stress. **A** Scheme summarizing the eukaryotic replisome complex coordinating DNA replication. DNA replication is carried out by specific DNA polymerases (Pol) on the leading and legging strands, respectively. Stretches of ssDNA are coated and stabilized by replication protein A (RPA). Unwinding of the DNA strands is carried out by minichromosome maintenance protein complex (MCM2-7) associated with cell division cycle 45 (CDC45) and go-ichi-ni-san (GINS) proteins. Altogether, they form the CMG complex. DNA replication is a process carried out with high fidelity. In order to accomplish this, eukaryotic cells express checkpoint proteins whose task is to closely monitor the genome and detect errors or problems during DNA replication. Checkpoint proteins are evolutionary well conserved and include ATR, ATRIP, CHK1, CLSPN, CDK2, Cdc7 which, altogether, mainly respond to stretches of ssDNA. Rad9–Hus1–Rad1 (9–1–1) is a heterotrimeric clamp on DNA sufficient to facilitate the interaction between ATR, its partner ATRIP, TOBP1 and CLSPN. During activation of a stress response following perturbation of DNA replication, CLSPN docks with CHK1 the major checkpoint effector kinase, contributing to promote the activation of surveillance mechanisms at the replisome. **B**, **C** Cells were incubated with vehicle or 1 μg/ml Dox for 72 h and then, where indicated, added 3 mM HU for 4 h (**B**), 6 h or 24 h (**C**). Harvested cells were processed as described in the materials and methods and whole cell lysates were analysed probing Western blot membranes with antibodies directed against proteins indicated in the figure. Experiments were performed at least three times obtaining similar results. β-Actin detection was used as loading control. One representative experiment is shown. Exp: exposure. Where indicated, the intensity of protein bands was quantified by densitometric analysis using ImageJ software (NIH) and calculated in percentage by assigning a value of 100% to specific bands as indicated in the figure

DNA replication starts at separate origins marked by the presence of ORC-dependent formation of pre-RCs. MCM2-7 protein complex is an important component of the pre-RC and it undergoes phosphorylation by cyclin E-CDK2 and Cdc7-Dbf4 protein kinases [51]. One of the major consequences of this concerted action is the loading of CDC45 which is essential for DNA unwinding and the binding of DNA polymerases to chromatin (Fig. 4A, [52]). MCM2 and MCM7 are direct targets of ATR checkpoint kinase and several lines of evidence suggest that the MCM2-7 complex is an important link between DNA replication and the DNA damage response to preserve genome stability in eukaryotic cells [53, 54].

To further elucidate the role of CK2α in DNA replication checkpoint, we determined the expression levels of several replication factors and proteins involved in checkpoint activation. Analysis of whole lysates from cells treated as indicated in Fig. 4B and C revealed that CK2α down-regulation in HU-treated cells not only impaired the ATR-mediated phosphorylation of CHK1 at S345 but also resulted in reduced expression of CLSPN (53% residual signal) and CDC45 (74% residual signal) as compared to control cells (Fig. 4B). The expression of Cdc7 remained unchanged under the applied experimental conditions. Moreover, lowered expression of CK2α resulted in significantly reduced levels of MCM3 in the absence (16% residual signal) and presence of HU treatment for 6 h (9% residual signal), respectively. Similar results were obtained in the case of MCM7 where we observed 71% and 51% residual signals in the absence and presence of HU treatment for 6 h, respectively, confirming also results from our previous investigations [22]. A similar outcome was observed in cells exposed or not to HU for 24 h (Fig. 4C). We reasoned that decreased phosphorylation of CHK1 at S345 could be a consequence of impaired ATR kinase activity. To verify this, we included the analysis of p53 phosphorylation at S15, which is an amino acid residue targeted by ATR in response to stress signals [55]. However, immunoblot analysis showed that the phosphorylation levels of p53 remained unchanged in cells treated with HU or a combination of HU and Dox. Although we cannot completely exclude the possibility that CK2α depletion negatively affects optimal kinase activity of ATR, results shown in Fig. 4B make it unlikely.

**Fig. 5:**
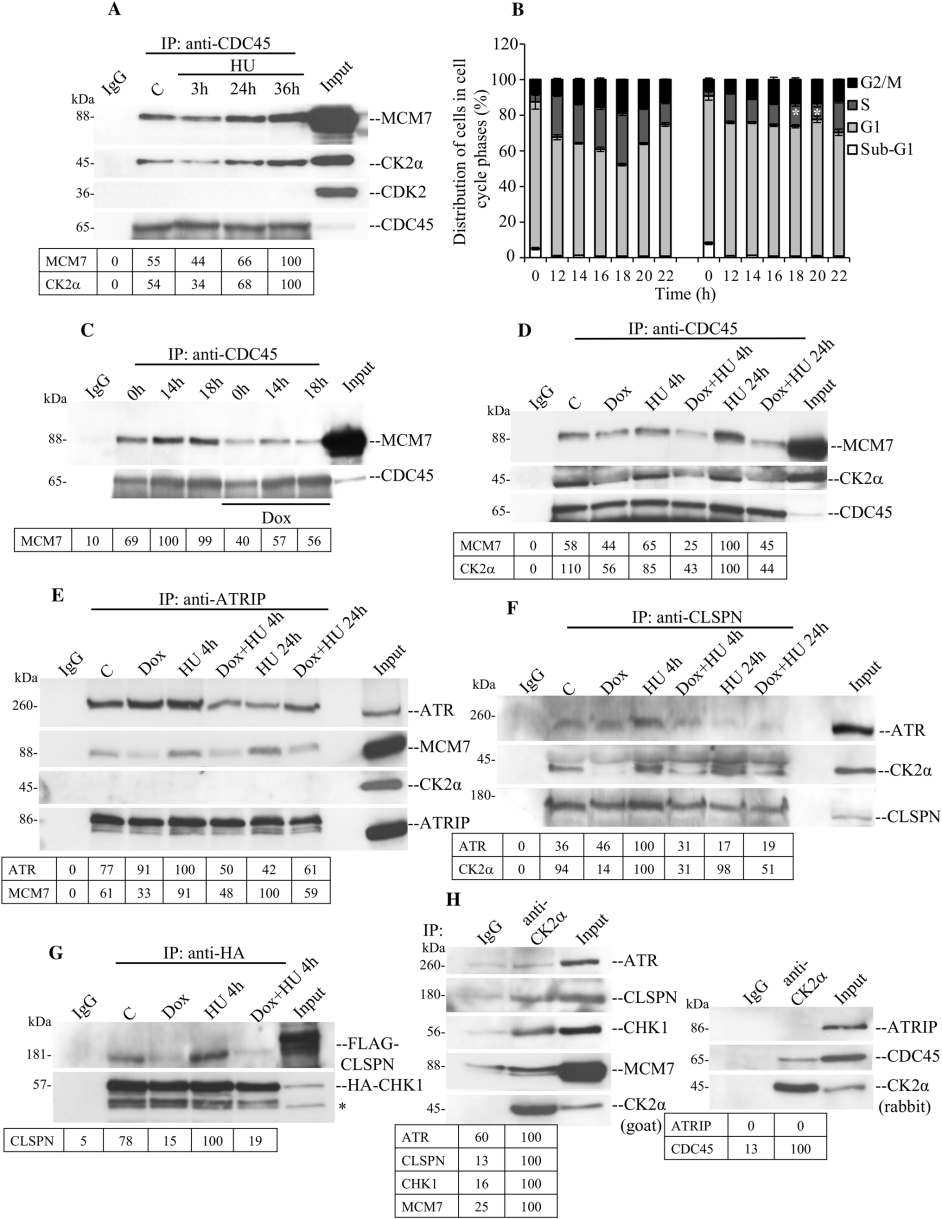
CK2α is required for DNA replication stress response. **A** H9c2-CK2α-44 cells were treated with vehicle or 3 mM HU for the indicated times. Whole cell lysates were subjected to immunoprecipitation (IP) with IgG (negative control experiment) or rabbit anti-CDC45 antibody. Samples were analysed by Western blot employing antibodies recognizing the proteins indicated in the figure. Whole cell lysate was also included in the analysis (Input) corresponding to 1% of the extract (control cells) employed in the immunoprecipitations. **B** FACS analysis of cells after synchronization by serum starvation (0.1% foetal bovine serum in the growth medium) for 48 h. Cells were released from cell cycle arrest when cultured again in the presence of complete growth medium. They were subsequently harvested at the indicated time-points and analysed. *P < 0.05 with respect to control cells at 18 h, and 20 h, respectively. **C** Cells were treated as indicated in **B** and in the presence or absence of Dox to induce down-regulation of CK2α. Whole cell lysates were subjected to immunoprecipitation with anti-CDC45 antibody. Samples were subsequently analysed as described in **A**. **D–F** Cells were left untreated or incubated with Dox for 72 h prior adding 3 mM HU for 4 and 24 h, respectively. Immunoprecipitation and Western blot analysis were then carried out as in **A** employing antibodies against the indicated proteins. **G** Cells were grown in the presence or absence of Dox for 72 h. After 24 h from the addition of Dox, cells were transfected with FLAG-CLSPN and HA-CHK1. 4 h before harvesting, cells were added HU as indicated in the figure. Immunoprecipitation and Western blot analysis were then carried out as described in **A**. **H** Whole cell lysates from H9c2-CK2α-44 cells were subjected to immunoprecipitation employing rabbit polyclonal anti-CK2α serum (results on the left side of the figure) or goat anti-CK2α antibody (results on the right side of the figure). Western blot analysis was subsequently carried out as in **A**. * Denotes non-specific band signal. All experiments were repeated at least three times obtaining similar results. Representative experiments are shown. Where indicated, the intensity of the protein bands was quantified by densitometric analysis as described in Fig. 4. Because of non-specific background signal in some results, the densitometric analysis of protein bands from the control experiments (IgG) is shown

Compelling evidence obtained with human and yeast cells has indicated that CDC45 has ssDNA binding affinity, may modulate DNA replication fork stalling and is likely to be an important target of the ATR-CHK1-mediated S-phase checkpoint [56–58]. It has been shown that CDC45 binds several proteins of the MCM2-7 complex including MCM7 [56, 58, 59], which is considered a critical regulator of the S-phase checkpoint [53]. Because of the effect on the expression levels of MCM2-7 proteins observed with CK2α down-regulation (Fig. 4C, [22]), it was of interest to determine whether the interaction between CDC45 and MCM7 is disrupted following induction of DNA replication stress and down-regulation of CK2α. As shown in Fig. 5A, MCM7 co-immunoprecipitated with CDC45 and the signal derived from the complex formation increased concomitantly with the length of exposure of cells to HU from 55% in control cells up to 100% in cells treated with HU for 36 h. We did not observe association with CDK2, consistent with the notion that CDK2 stimulates the ATR-CHK1 pathway and is required for an efficient DNA replication checkpoint response by catalysing the phosphorylation of specific substrates rather than associating with its target proteins [60]. Interestingly, we found that also the signal from the detected CK2α associated with MCM7 and CDC45 was increased under these experimental conditions.

The increased binding between MCM7 and CDC45 observed in cells treated for up to 36 h with HU suggested that this effect could be dependent on the gradual accumulation of cells in S-phase. To verify this, cells were synchronized by serum starvation for 48 h and, upon stimulation with full growth medium, collected at different time intervals as indicated in Fig. 5B. Next, we verified the association between CDC45 and MCM7 employing whole extracts from cells harvested at 0 h, 14 h and 18 h, respectively. Immunoblot analysis revealed that the association between CDC45 and MCM7 increased during S-phase confirming findings reported previously [61]. Most importantly, down-regulation of CK2α caused a significant decrease in the signal relative to the interaction between CDC45 and MCM7 (Fig. 5C). Next, to determine the role of CK2α in the complex formation between MCM7 and CDC45 following HU treatment, we carried out co-immunoprecipitation experiments as shown in Fig. 5D. The signal relative to the association between CDC45 and MCM7 was slightly higher after 4 h of incubation with HU (58% signal in control cells and 65% in cells exposed to HU for 4 h as compared to cells treated with HU for 24 h) and it further increased after 24 h of incubation. Conversely, the signal derived from the detection of these proteins was significantly decreased in cells with reduced levels of CK2α (i.e. 25% and 45% residual signal after treatment with HU for 4 h and 24 h, respectively).

Overall, these results show that CK2α is required for efficient interaction between CDC45 and MCM7 during DNA replication and S-phase checkpoint activation (Fig. 8).

Optimal phosphorylation of CHK1 by ATR requires the coordinated interaction of several replication and checkpoint proteins. MCM7 is required for ATR foci formation by interacting with ATRIP, thus, facilitating ATR tethering to the replication fork and optimal CHK1 phosphorylation in response to ssDNA [53, 62]. Based on this, we determined whether the interaction between MCM7 and ATRIP was affected concomitantly with down-regulation of CK2α and HU treatment. Analysis of immunoprecipitates by Western blot, revealed increased association between MCM7 and ATRIP in cells treated with HU and harvested after 4 h and 24 h, respectively, as compared to control experiments (Fig. 5E). This confirms previous results demonstrating that the association of MCM7 with ATRIP is enhanced in replication checkpoint signalling [62]. Conversely, detection of this complex formation was found significantly reduced in cells with down-regulated CK2α as indicated by the densitometric analysis of protein band signal (Fig. 5E). In line with previous findings, we confirmed stable association between ATR and ATRIP in control cells and no evidence of altered ATR–ATRIP complex formation in response to HU treatment [5, 63–65]. Conversely, association between ATRIP and CK2α was not observed under the applied experimental conditions (Fig. 5E). Collectively, these results suggest that MCM7 is closely associated with the ATR–ATRIP complex and although a direct association between ATRIP and CK2α could not been observed, our results suggest that down-regulation of CK2α might negatively impact the stability of the ATR–ATRIP–MCM7 complex formation (Fig. 8).

Different lines of evidence indicate that ATR phosphorylates and might constitutively interact with the adaptor protein CLSPN enabling it to bind CHK1, thus, facilitating ATR-mediated phosphorylation of the effector kinase [58, 66, 67]. Hence, we asked whether endogenous CLSPN associates with ATR, CHK1 and possibly CK2α by performing immunoprecipitation experiments employing anti-CLSPN antibodies. We identified a weak but detectable interaction of endogenous CLSPN with ATR in control cells and cells treated with HU for 4 h. However, the signal derived from their complex formation was not detectable at 24 h of incubation with HU (17% residual signal for ATR detection) nor in cells with down-regulated CK2α (19% residual signal for ATR detection, Fig. 5F). Most importantly, we show for the first time that CK2α is precipitated by anti-CLSPN antibodies and that its down-regulation seems to severely compromise the association between CLSPN and ATR (Fig. 5F). Although we readily observed binding of CLSPN to ATR and CK2α, we could not detect a complex between CLSPN and endogenous CHK1 under the applied experimental conditions. As previous studies suggested such a physical interaction between CLSPN and CHK1, we asked whether lack of detection of this complex in myoblasts could be due to technical limitations. To investigate this, we co-transfected cells with plasmids expressing FLAG-CLSPN and HA-CHK1 and carried out immunoprecipitation experiments with anti-HA antibodies. As shown in Fig. 5G, we readily detected association between FLAG-CLSPN and HA-CHK1, which slightly increased upon 4 h of incubation with HU. Interestingly, decreased expression of CK2α resulted in significantly decreased detection levels of their interaction both in the absence and presence of replication stress.

In subsequent experiments employing whole lysate from control cells, we demonstrated that anti-CK2α antibodies co-precipitated endogenous CLSPN, CHK1, CDC45 and MCM7 and not ATR or ATRIP (Fig. 5H). Collectively, these results show that CK2α associates with essential components of the replisome, which are critical factors in DNA replication and in the activation of checkpoint signalling in response to stalled replication forks (Fig. 8).

To validate key findings reported above with different cells we, additionally, analysed the response to HU treatment following siRNA-mediated silencing of CK2α, in human cells. For this, we employed U2OS cells and incubated them with HU for increasing lengths of time. Flow cytometry-based analysis of the cells showed accumulation at G1/S-phase as early as 6 h after adding the compound and up to 24 h of incubation with HU (Suppl. Fig S5A). Thereafter, the cell cycle was resumed. Next, we transfected the cells with CK2α-siRNA and treated them with 3 mM HU for 4 h. Cell extracts were employed for immunoprecipitation experiments in the presence of anti-CDC45 antibodies (Suppl. Fig S5B). MCM7 co-immunoprecipitated with CDC45 and CK2α and the amount of MCM7 associated with CDC45 increased in HU-treated cells. Conversely, in CK2α-depleted cells, the amount of MCM7 interacting with CDC45 significantly decreased in cells left untreated or only incubated with HU. This indicates that MCM7–CDC45 interaction is dependent on the presence of CK2α as observed in myoblasts (Fig. 5D). In experiments conducted with myoblasts, we observed interaction between CLSPN and ATR (Fig. 5F). When we investigated their association in U2OS cells, however, we were able to detect a complex between CLSPN and CK2α but not between CLSPN and ATR (Suppl. Fig S5C). We additionally conducted reciprocal immunoprecipitation experiments essentially as shown in Fig. 5H using anti-CK2α antibodies. Experiments yielded results consistent with those shown in Fig. 5H, confirming that also in U2OS cells, CK2α interacts with CLSPN, CHK1, CDC45 and MCM7 (Suppl. Fig S5D).

### CK2α is important for the recruitment and/or stability of CLSPN and CDC45 to chromatin during induction of checkpoint signalling

**Fig. 6:**
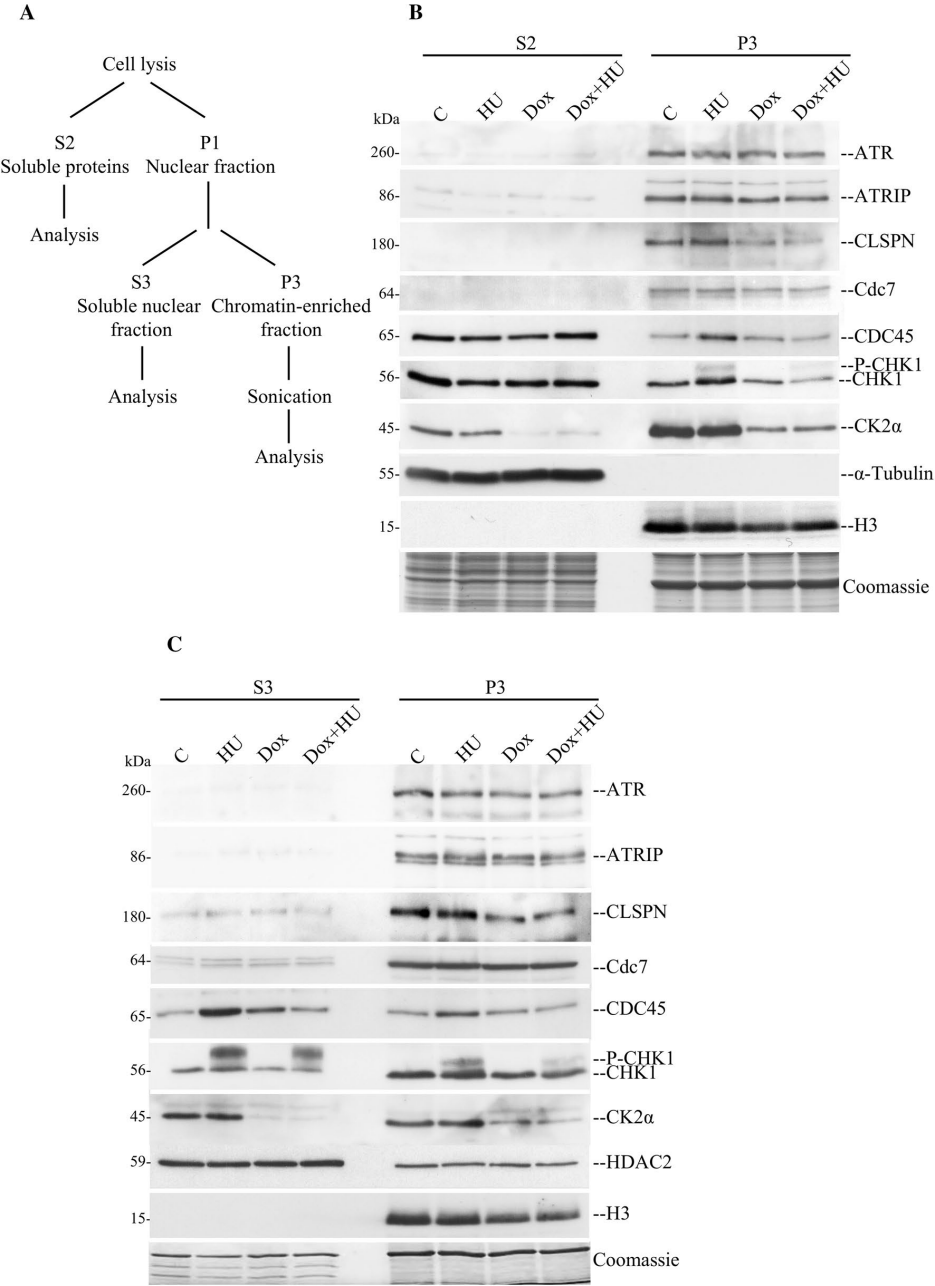
Effect of CK2α down-regulation on chromatin binding of DNA replication factors. **A** Cells were left untreated or incubated with 1 μg/ml Dox for 72 h prior adding 3 mM HU for 4 h. The resulting cultures were subsequently separated into various fractions as shown in the scheme of the biochemical fractionation method. **B**, **C** The different fractions were separated by SDS-PAGE. Separated proteins were analysed by either Coomassie staining, which served as a loading control, or Western blot by probing the membranes with antibodies directed against the proteins indicated in the figure. Detection of α-tubulin served as a control as this protein is expressed in the cytoplasm of cells during interphase [76]. Coomassie stained gels show equal loading of proteins derived from the different fractions isolated with the protocol applied for crude subcellular fractionation of cultured mammalian cells

In response to replication block, ATR and CLSPN are both required for optimal activation of CHK1 [7, 68, 69]. Under these conditions, CHK1 is recruited to sites of DNA damage in a CLSPN-dependent manner where it is phosphorylated by ATR [11]. Furthermore, it has been established that CHK1 binds to chromatin in unperturbed cells but dissociates from it to phosphorylate downstream effectors in response to DNA damage [70–72]. To investigate the cellular localization of CK2α’s interaction partners following induction of replication stress, we fractionated whole cell lysates. We separated cytoplasmic proteins (S2) from soluble nuclear proteins (S3) and a chromatin-enriched fraction (P3, Fig. 6A) essentially as described in [36]. Western blot analysis of these different fractions revealed that ATR, ATRIP and Cdc7 were largely detected in the chromatin-enriched fraction supporting the notion that these proteins are primarily associated with chromatin. However, no differences in their binding were seen under the applied experimental conditions (Fig. 6B and C). This is consistent with the hypothesis that at least in the case of ATR and ATRIP, the level of recruitment to chromatin does not increase following induction of DNA replication stress. These proteins might, instead, undergo a change in the mode by which they bind to chromatin as previously suggested [64]. This possibility should not be excluded also in the case of Cdc7 where a marginally increased binding to chromatin has been reported in experiments with HU and chromatin isolation in low salt conditions [73, 74]. Next, we investigated the expression of CLSPN in soluble nuclear and chromatin-enriched fractions (Fig. 6C). We found that the majority of CLSPN was associated with chromatin in control cells and the amount did not change in response to HU as previously reported [74]. Conversely, the levels of CLSPN in the P3 fraction decreased significantly in cells with down-regulated CK2α and in the absence or presence of HU. In agreement with previous studies [1], CDC45 was detected in all three fractions. Part of this protein localized in the S3 and P3 fractions in response to HU. However, as observed for CLSPN, the levels of CDC45 in the soluble nuclear and chromatin-enriched fractions decreased considerably when CK2α was down-regulated (Fig. 6C). Analysis of endogenous CHK1 revealed that this effector kinase was present in the cytoplasmic, nuclear and chromatin fractions as previously described [72, 75] whereas the phosphorylated form was more abundant in the soluble nuclear fraction than in the P3 fraction in cells treated with HU. This supports the notion that CHK1 can bind chromatin in unperturbed cells but rapidly dissociates from it in response to DNA damage to facilitate transmission of the DNA damage signal to downstream target proteins [72, 75]. Interestingly, the amounts of CHK1 and phosphorylated CHK1 in the S3 and P3 fractions were decreased in cells with down-regulated CK2α (Fig. 6C). As an important control for the fractionation method applied in our investigations, we exclusively observed expression of α-Tubulin in the S2 fraction and not in the P3 fraction (Fig. 6B) making unlikely a cross contamination between soluble proteins present in the S2 fraction and our nuclear preparations.

Overall, these results indicate that CK2α is required for maintaining proper association of CLSPN, CDC45 and CHK1 with chromatin and/or that this kinase is essential for preserving their stability both in unperturbed and replication stress-induced conditions.

### Cells with reduced expression of CK2α show delayed stalled fork recovery and defects in S-phase checkpoint in response to deprivation of deoxynucleotides

**Fig. 7:**
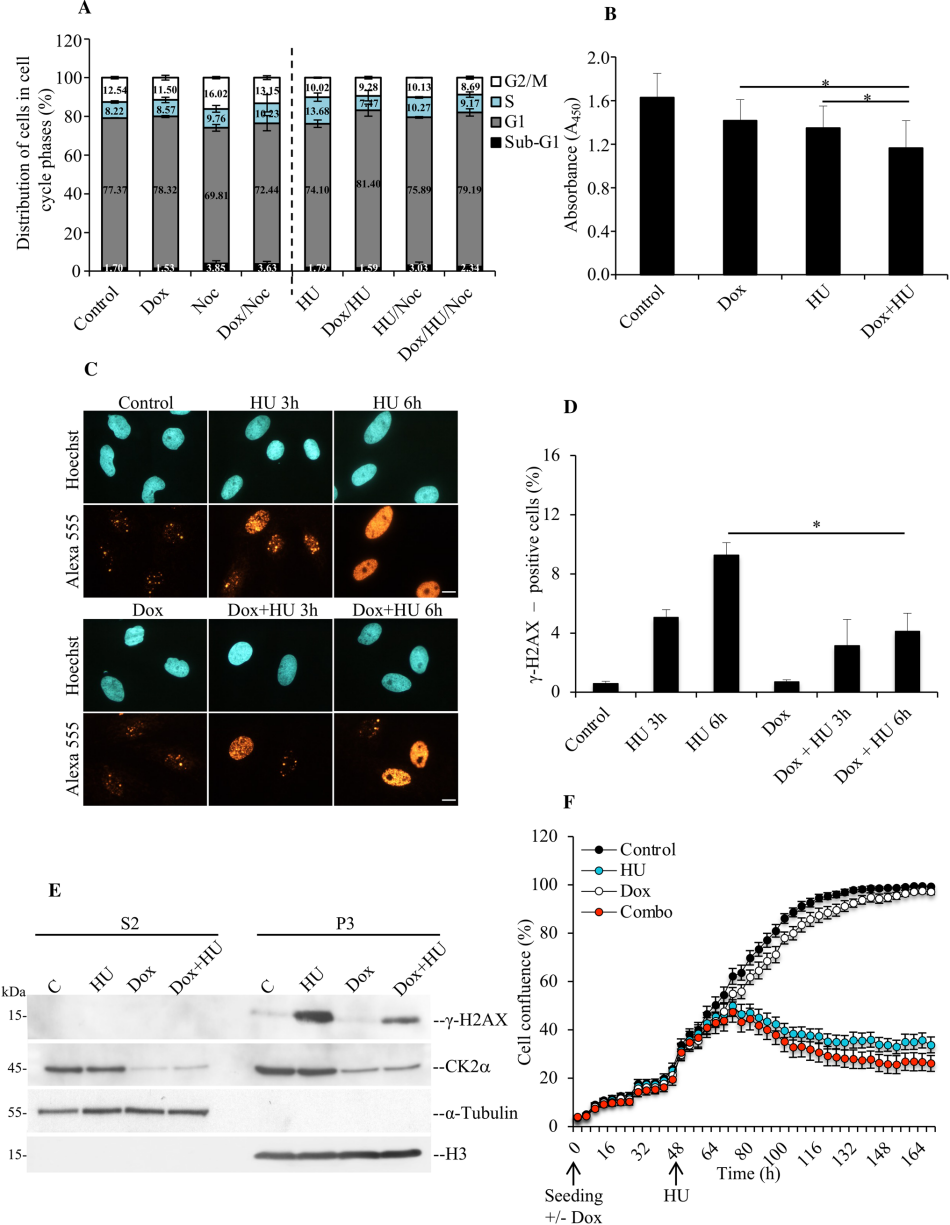
Cells depleted of CK2α show delayed entry into S-phase and compromised recovery from cell cycle arrest following HU treatment. **A** Cells left untreated or exposed to Dox for 72 h were added 3 mM HU in the last 12 h of incubation time. 6 h before harvesting, cells were trapped in mitosis by adding 0.2 μg/ml nocodazole (Noc). Harvested cells were subsequently processed for flow cytometry analysis. Bar graph shows percentage of cells in the various phases of the cell cycle according to their DNA content. **B** Detection of incorporated BrdU was performed by a colorimetric assay. Cells were treated essentially as in **A**. After 12 h of HU treatment, cells were washed and allow to recover in fresh growth medium containing 10 μM BrdU for 10 h. Detection of fixed cells was carried out by staining cells with an anti-BrdU antibody coupled to horseradish peroxidase. Colorimetric reactions were quantified by measuring the absorbance at 450 nm. Average values (*n* = 10 replicates) ± standard deviation are shown in arbitrary units, *P < 0.05. **C**, **D** Cells were treated essentially as described in Fig. 2B. Immunofluorescence of cells stained with an antibody to γ-H2AX is shown. Quantification of Alexa 555-positive cells was performed by manual counting and expressed as percentage of the total number of cells in each sample revealed by nuclei staining with Hoechst 33258 (D). Photos shown in the figure were taken at 40 × magnification. Scale bar represents 20 μm. Bars on the graph indicate mean values ± standard deviation from three independent experiments. Asterisk denotes statistical significance: *P < 0.05. **E** Cell fractions prepared as described in Fig. 6 were analysed by Western blot employing antibodies against H3, CK2α and phosphorylated H2AX (γ-H2AX), respectively. **F** Cells were plated in 96-well plates in the presence or absence of doxycycline (± Dox). After 48 h and where indicated, cells were added 3 mM HU until the termination of the experiments. Cell growth was analysed by the IncuCyte S3 live-cell system. Measurements were performed every 4 h. Data represent mean values ± standard deviation (*n* = 10 replicates) and expressed in percentage. Results shown were repeated three times obtaining similar results

To evaluate the ability of the cells to recover from replication block induced in the presence of HU, myoblasts left untreated or exposed to Dox for 72 h were incubated with 3 mM HU in the last 12 h of incubation time. Six hours prior to harvesting, cells were trapped in mitosis by adding 0.2 μg/ml nocodazole (Noc). As expected, the analysis of cells by flow cytometry showed that 16.02% of control cells treated with nocodazole accumulated in G2/M-phase whereas additional down-regulation of CK2α resulted in 13.15% accumulation of cells in G2/M-phase (Fig. 7A). This supports the notion that cells with down-regulated CK2α expression display delayed cell cycle progression as previously observed [22, 34].

Exposure to HU resulted in 10.02% accumulation of cells in G2/M-phase whereas additional treatment with nocodazole led to a 10.13% accumulation of cells in G2/M-phase. Cells with down-regulation of CK2α displayed a slightly, but reproducible, decreased number of cells in G2/M-phase (9.28% in the absence of nocodazole and 8.69% in the presence of it, respectively). This suggests that cells with down-regulated expression of CK2α undergo a prolonged arrest in G1/S-phase and/or have difficulties to resume cell cycle progression after fork arrest in the presence of HU. To verify this, we investigated the rate of cellular DNA synthesis by measuring BrdU incorporation in control and CK2α-depleted cells, respectively, (Fig. 7B). We treated cells with HU for 12 h and then measured BrdU incorporation after the removal of HU. In the absence of HU, fewer cells were in S-phase after CK2α down-regulation suggesting a slower proliferation rate. After treatment with HU, CK2α-down-regulated cells had a significantly reduced BrdU incorporation as compared to cells incubated with HU alone. This suggests that CK2α is important for S-phase checkpoint recovery following treatment with HU and underlines the functional role of this protein kinase in maintaining replication potential during unperturbed DNA replication and in response to replication stress. We cannot, however, exclude the possibility that this protein kinase might, additionally, control cell cycle progression through S-phase.

The histone variant H2AX has been implicated in the maintenance of genomic stability in response to induction of DNA double-strand breaks (DSBs) signalled by its phosphorylation catalysed by a phosphatidylinositol 3-OH-kinase-related kinase, mainly ATM [1, 77]. However, compelling evidence has indicated that H2AX is also phosphorylated in an ATR-dependent manner in response to stalled fork formation making it a sensitive indicator of both DNA damage and DNA replication stress [77, 78]. Based on this, we asked whether down-regulation of CK2α affects the phosphorylation levels of H2AX. In this respect, it has been shown that CHK1 inhibition or its siRNA-mediated down-regulation causes formation of ssDNA with increased levels of RPA bound to chromatin, which triggers rapid ATR-mediated phosphorylation of H2AX (γ-H2AX) in exponentially growing cells [79]. Hence, to test whether down-regulation of CK2α is associated with changes in the levels of γ-H2AX, we stained cells with anti-phospho-H2AX (S139, Fig. 7C) and quantified the signal as indicated in Fig. 7D. Exposure of cells to HU for 3 h and 6 h, respectively, resulted in increased pan-nuclear phosphorylation of H2AX following replication stress induction as compared to control cells. Unexpectedly, down-regulation of CK2α caused a significant reduction in γ-H2AX signal suggesting compromised signalling to the checkpoint machinery. In support of results shown in Fig. 7C and D, we additionally performed Western blot analysis of chromatin-enriched protein fractions (Fig. 7E) and super-resolution microscopy (QIBC) analysis of cells treated as indicated in Suppl. Fig S6 and immunolabelled with anti-γ-H2AX antibody to mark the phosphorylation of H2AX during the cell cycle. As shown in Fig. 7E and Suppl. Fig S6, γ-H2AX signal was significantly lower in cells with reduced levels of CK2α and exposed to HU as compared to cells incubated with HU alone unequivocally indicating that down-regulation of CK2α prevents phosphorylation of H2AX in response to DNA replication stress.

The newly discovered connection between CK2α and DNA replication stress response prompted us to also examine the effect on cell proliferation. We monitored proliferation at extensive lengths of time employing the IncuCyte S3 live-cell analysis system. Figure 7F shows that cells incubated with HU alone displayed a significant reduction in the proliferation rate as compared to control cells. When CK2α expression was also reduced, cells responded with a further reduction in proliferation with respect to control cells and those treated with Dox or HU, respectively.

Taken together, results shown in this study provide compelling evidence that cells with reduced levels of CK2α do not progress from G1 to S-phase at the same pace when subjected to HU-induced replication stress. Our data suggest that CK2α plays a specialized role in preserving replication fork integrity by ensuring the stable association of specific replication fork factors. Consequently, cells with reduced levels of CK2α experience (i) less efficient or delayed entry into S-phase, (ii) compromised S-phase checkpoint activation in response to DNA replication stress and (iii) defective recovery of stalled replication forks at the cost of cell growth.

## Discussion

In this study, we have identified a novel function of CK2α as a protein kinase essential in DNA replication checkpoint signalling. Depletion of CK2α correlates with a significant reduction in the phosphorylation of CHK1 suggesting impaired activation of the ATR-CHK1 signalling cascade. In response to replication stress, ATR activation results in its recruitment to RPA-coated ssDNA in a process which depends on its interaction partner ATRIP. From the analysis of p53 phosphorylation at S15 we concluded that ATR kinase activity per se remains unaltered in cells depleted of CK2α. However, we cannot exclude the possibility that optimal activation of ATR cannot be reached in cells depleted of CK2α as this event is a multi-step process involving several checkpoint proteins. In this respect, one of the best characterized activators of ATR is TopBP1 which contains an ATR-activation domain that stimulates ATR kinase activity [80]. Interestingly, CK2 has been shown to regulate the stability of TopBP1 by phosphorylating histone demethylase PHF8 which binds TopBP1 preventing its degradation to maintain genome stability [81]. Hence, the involvement of TopBP1 in the CK2α-mediated regulation of ATR-CHK1 signalling axis is an attractive possibility that warrants further work, in the future.

Among the array of factors present at the replication fork that contribute to the cellular response to DNA replication stress, we have identified several proteins regulated by CK2α. CLSPN, a checkpoint clamp loader, is necessary for ATR-dependent activation of CHK1 [66]. We found that signal derived from its association with ATR is significantly impaired in cells with down-regulation of CK2α (Fig. 5F). This could be due to CLSPN’s reduced expression under experimental conditions indicated in Figs. 4 and 6. However, the ATR-CHK1 signalling pathway is subjected to several layers of regulation and while a complex has been detected between human CLSPN and ATR, it has not to be excluded that other proteins such as TopBP1 [12] and AND-1 [82], which have been shown to bind ssDNA and be important regulators for efficient CHK1 activation, could be subjected to CK2α regulation as well. Additionally, CLSPN expression is regulated by the proteasome-mediated degradation machinery and down-regulation or inhibition of CHK1 have been found to decrease CLSPN stability implying a reciprocal regulation during the cell cycle [83]. CK2α-mediated phosphorylation of CLSPN could also contribute to maintain its protein stability as CLSPN’s amino acid sequence displays several putative CK2-dependent phosphorylation sites. We attempted to verify CLSPN-ATR binding also in U2OS cells. However, we could not demonstrate their association, in accordance with previous studies [66]. This reinforces the notion that their complex formation can be transient and that there might be some differences in the strength of their binding among cell types. Nonetheless, our data suggest that both CLSPN and CK2α are required to sustain CHK1 activation during DNA replication stress response and that reduced levels of CK2α may destabilize this complex formation resulting in accelerated degradation of CLSPN and impaired activation of CHK1.

Interestingly, we found that CK2α associates with CHK1. We previously showed by gel filtration analysis combined to immunoprecipitation assays that CHK1 mainly interacts with the CK2β isoform of protein kinase CK2 and only marginally with CK2α in cancer cells [27]. Cells with reduced levels of CK2α display decreased CK2 kinase activity and reduced expression levels of CK2β as previously observed (Fig. 1C, D, [22]). Whereas the contribution of CK2β to the association between CHK1 and CK2α in myoblasts remains to be investigated, it is not to be excluded that the presence of CHK1 in anti-CK2α immunoprecipitates might also be due to the presence of another replisome factor co-precipitating with CK2α.

The correct regulation of CHK1 also depends on other proteins such as MCM7. This helicase has been shown to be a direct target of the ATR-mediated signalling cascade making it an attractive candidate linking the DNA replication machinery to checkpoint activation in S-phase (reviewed in [11]). We demonstrated that CK2α co-precipitates with MCM7 and its co-factor CDC45 and their complex formation increases as cells accumulate in S-phase. This suggests that CK2α is required for replication initiation and, possibly, the elongation step of DNA replication. Moreover, we show that signal relative to the association between MCM7 and CDC45 is significantly decreased in cells with reduced levels of CK2α. While this indicates that CK2α could contribute to stabilize their interaction we cannot exclude that lack of detection of this complex formation may result from decreased expression levels of MCM7 in cells with down-regulated CK2α. This was previously reported by us in in vitro and in vivo investigations [22] and it is consistent with the possibility that CK2α might directly or indirectly protect MCM7 from proteasome-mediated degradation. In support of this, Buchsbaum et al. [84] showed that endogenous MCM7 can be polyubiquitinated and that proteasome-mediated inhibition increases the intracellular amounts. Regulation of MCM7 expression as well as the other members of the MCM2-7 complex has not been fully investigated and can occur at multiple levels. Chuang et al. [85] identified two new mechanisms that regulate MCM proteins expression showing that the stoichiometry of the MCM components is controlled post-transcriptionally at both mRNA and protein levels. Interestingly, Montagnoli et al. [86] showed that CK2 phosphorylates MCM2 at a highly conserved canonical consensus sequence. Hence, it is not to be excluded that MCM7 might as well serve as a substrate for protein kinase CK2. Hence, future experiments will be pivotal for understanding how CK2α controls the expression of eukaryotic MCM helicases. This knowledge will provide further insights into the mechanism by which CK2α regulates DNA replication under normal as well as perturbed conditions affecting S-phase.

Biochemical fractionation separating soluble from chromatin-bound proteins showed that CDC45 is present in all three fractions as reported in previous studies [1, 59, 61]. Whereas the cytosolic fraction of CDC45 remained unchanged under experimental conditions shown in Fig. 6B and C, we reported evidence of CDC45 accumulation in the nucleoplasm and in the fraction of proteins associated with chromatin following incubation with HU. This pattern of expression of CDC45 in the nuclear fraction changed upon down-regulation of CK2α. This raises several possibilities that could potentially explain the decrease in CDC45 expression in cells with reduced levels of CK2α including (i) dissociation of CDC45 from chromatin, (ii) its enhanced degradation and/or (iii) altered expression mediated by a transcription factor. It is unlikely that lack of CK2α causes detachment of CDC45 from chromatin as we do not observe a concomitant increase of signal in the nucleoplasm or cytosol. We cannot, however, exclude the possibility of increased degradation. CDC45 contains several PEST and KEN domains that facilitate its breakdown [87] and this might suggest that its half-life is just sufficient to cover one S-phase [88]. The possibility that a transcription factor might be negatively affected in cells with reduced levels of CK2α is an attractive alternative. CDC45, but also MCM proteins, have been shown to be under the control of E2F transcription factors [89–93] through mechanisms in part dependent on the activation of cyclin E-CDK2, which is one of the major upstream regulators of the retinoblastoma (Rb)/E2F complex [89]. Interestingly, we previously demonstrated that cells with down-regulated expression of CK2α display significantly reduced kinase activity of the cyclin E-CDK2 complex and concomitantly, inhibition of E2F transcriptional activity [22, 34]. Finally, CDK2 acts in concert with Cdc7 to facilitate loading of CDC45 onto the DNA [94]. We have not observed differences in the amount of Cdc7 associated with chromatin in the presence or absence of CK2α, however, this does not exclude the possibility that CK2α might regulate the kinase activity of Cdc7. In this respect, CK2α has been shown to phosphorylate nuclear phosphoglycerate kinase (PGK) 1 kinase resulting in interaction of PGK1 and Cdc7 and promoting the recruitment of DNA helicases to replication origins [95].

**Fig. 8:**
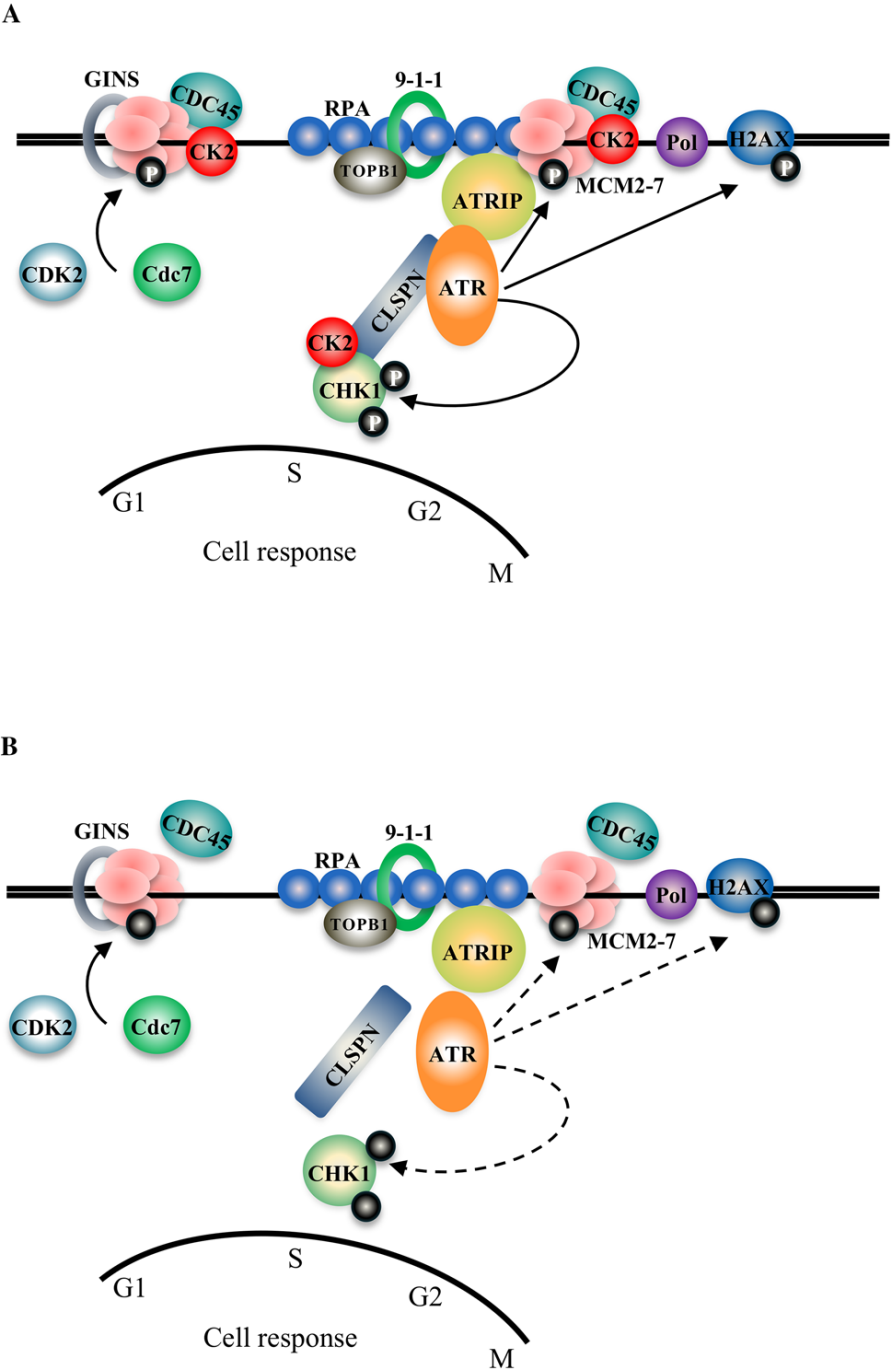
Model of CK2α-mediated regulation of cell cycle checkpoint response following induction of DNA replication stress. **A** Multiple replication factors contribute to regulate the DNA replication stress response preventing DNA damage and allowing completion of DNA replication. The slowing down or stalling of replication forks exposes ssDNA stretches which attract RPA whose function is to coat and stabilize these segments of DNA. It follows the recruitment of ATR–ATRIP complex which, in turn, phosphorylates CHK1 activating the checkpoint signalling. CLSPN interacts with CHK1 contributing to efficient ATR-mediated phosphorylation and activation of CHK1. We provide evidence showing that CK2α interacts with both CLSPN and CHK1 and stabilizes their association. The replisome is connected to DNA replication stress checkpoint machinery. Evidence shows that several protein kinases including Cdc7, CDK2 and ATR, phosphorylate various components of the MCM protein complex. In particular, ATM/ATR-dependent MCM phosphorylation primarily responds to replication stress. Besides CLSPN, efficient phosphorylation of CHK1 is mediated by other proteins including MCM7, RAD17, TopBP1 and the MRN complex. We found that CK2α interacts with MCM7 and CDC45 contributing to their assembly and localization on chromatin during normal cell cycle and upon induction of DNA replication stress, respectively. **B** We propose that CK2α plays a key role in maintaining the integrity of the replisome by stabilizing the association of specific replication factors and ensuring optimal activation of the ATR-CHK1 checkpoint pathway upon induction of replication stress. Down-regulation of CK2α leads to destabilization of the aforementioned complex formations which results in compromised DNA checkpoint response

Apart from CHK1, ATR phosphorylates other checkpoint proteins at stalled replication forks including the histone variant H2AX at S139 [11]. This occurs mostly in early S-phase [96]. Interestingly, we observed that H2AX was not phosphorylated in cells with down-regulation of CK2α. Given the effect on essential replication factors, we hypothesized that cells with down-regulated CK2α and exposed to HU would display elevated levels of spontaneous DNA damage and elevated γ-H2AX signal. This was, however, not the case (Fig. 7). In line with our findings, Syljuåsen et al. [79] showed that phosphorylation of H2AX is prevented in cells treated with CDK inhibitors and shRNA-mediated down-regulation of CDK2, respectively, in response to CHK1 inhibition. Moreover, Gagou et al. [78] reported evidence that H2AX phosphorylation occurs at sites of stalled forks and that this is strictly dependent on the presence of CDC45. Although we did not detect a significant change in the viability of the cells, results shown in Fig. 7 indicate, altogether, that cells are failing to recover from HU-induced arrest and slowly lose proliferation potential when CK2α is down-regulated. Since phosphorylation of histone H2AX is implicated in the maintenance of DNA stability, decreased accumulation of γ-H2AX could just be the consequence of incorrect assembly and/or expression of DNA repair complexes and compromised checkpoint signalling (Fig. 8).

## Conclusion

Overall, results presented in this study provide novel insights into critical mechanisms which control DNA synthesis and activation of DNA replication checkpoint and suggest that CK2α is a key player in these processes by ensuring the stability and/or association of protein factors present at DNA replication forks. Most importantly, altered expression of CK2α could sensitize cells to additional DNA damaging stresses, induce aberrant cell cycle progression and augment cell death.

### Supplementary Information

Below is the link to the electronic supplementary material.

Supplementary file1 Suppl. Fig S1. Expression pattern of phosphorylated CHK1 and H2AX in cells - negative controls. **A** Negative control (NC) for phospho-CHK1 (S345) staining of myoblasts cells. Images were taken at 20x magnification. Scale bar represents 50 μm. **B** Negative control for phospho-H2AX (S139) staining of myoblasts cells. Images were taken at 40x magnification. Scale bar represents 20 μm (TIFF 851 KB)

Supplementary file2 Suppl. Fig S2. Response of myoblasts to treatment with aphidicolin or exposure to UV irradiation. **A**, **C** Cells were treated with 5 μM aphidicolin (Aph) to inhibit DNA polymerase (A) or exposed to 60 J/m2 UV irradiation (C) to induce DNA replication stress [49]. Cells were harvested at different time points and analysed by Flow cytometry after staining the DNA with propidium iodide. *P < 0.005 with respect to control cells at 24 h, and 36 h, respectively (A), *P = 0.05 with respect to control cells at 12 h and ^#^*P* < 0.005 with respect to control cells at 24 h (C). **B**, **D** Western blot analysis of whole lysates from cells treated essentially as indicated above, was carried out employing antibodies against proteins indicated in the figure. β-actin detection served as loading control. (TIFF 3334 KB)

Supplementary file3 Suppl. Fig S3. Gene expression changes within the cell cycle pathway following treatment of H9c2-CK2α-44 with Dox, HU, or a combination. Following treatment of cells with either 1 μg/ml Dox for 72 h, 3 mM HU for 24 h or a combination, RNA-sequencing was used to obtain log2 fold-change estimates of gene expression changes relative to untreated controls. The changes in gene expression are indicated in colour, with bright red indicating a positive log2 fold-change of at least 2, and bright blue a negative log2 fold-change of at least 2. The three conditions are displayed such that for each gene or gene-group, the first colour shows the change in gene expression following Dox treatment alone, the second colour the combination of Dox and HU, and the last colour the effect on gene expression following treatment with HU alone.(TIFF 1247 KB)

Supplementary file4 Suppl. Fig S4. Gene expression changes relative to proteins previously examined by Western blot. Cells were treated as indicated in Suppl. Fig S3. RNA-sequencing was used to obtain normalized expression estimates of genes coding for proteins examined by Western blot. The four different conditions are indicated by colour as indicated in the figure. Gene expression estimates were log10 transformed to plot highly differing gene expression estimates within the same plot.(TIFF 1344 KB)

Supplementary file5 Suppl. Fig S5. Analysis of complex formations in U2OS cells following down-regulation of CK2α and DNA replication stress induction. **A** U2OS cells were treated with 3 mM HU and harvested at the indicated time points. *P < 0.005 with respect to control cells at 6 h, 12 h, and 24 h, respectively. Experiments were subsequently carried out as described in Fig 1A. **B**, **C** Cells were transfected for 56 h with CK2α-siRNA to induce down-regulation of the protein kinase. It followed incubation with 3 mM HU for additional 4 h before harvesting. Whole cell lysates were subsequently subjected to immunoprecipitation assays essentially as described in Fig 5 with anti-CDC45 (B) and anti-CLSPN antibodies (C), respectively. **D** Whole cell lysate from U2OS cells was employed in immunoprecipitation studies as described in Fig 5H. The identification of the co-precipitated proteins was carried out by Western blot employing antibodies indicated in the figure. In the analysis of the immunoblots, we noticed that the detection of MCM7 resulted in two band signals corresponding to proteins with distinct molecular weights suggesting co-immunoprecipitation of full length and a shorter form of MCM7. This is plausible as alternatively spliced transcript variants encoding distinct isoforms of MCM7 have been reported (http://atlasgeneticsoncology.org/Genes/GC_MCM7.html). (TIFF 1939 KB)

Supplementary file6 Suppl. Fig S6. Analysis of cells by quantitative image-based cytometry (QIBC) reveals decreased levels of phosphorylation of H2AX in cells with down-regulation of CK2α and exposed to HU. **A** QIBC analysis of cells exposed to 3 mM HU for the indicated times and immunostained for γ-H2AX. Nuclear DNA was counterstained by 4′,6-diamidino-2-phenylindole (DAPI), n >4,000 cells for each condition. The colour gradient indicates the mean nuclear γ-H2AX intensity. A.U., arbitrary units. **B** Quantification of γ-H2AX signal intensity from the experiment performed in A. The data points represent average values. n > 4,000 cells. Statistical analysis were done with GraphPad Prism (GraphPad Software version 9) using one-way ANOVA. (TIFF 8428 KB)

## Data Availability

RNA-seq data have been deposited in the ArrayExpress database at EMBL-EBI under accession number: E-MTAB-10752.

## References

[1] Aparicio T, Ibarra A, Mendez J (2006). Cdc45-MCM-GINS, a new power player for DNA replication. Cell Div.

[2] Moyer SE, Lewis PW, Botchan MR (2006). Isolation of the Cdc45/Mcm2-7/GINS (CMG) complex, a candidate for the eukaryotic DNA replication fork helicase. Proc Natl Acad Sci USA.

[3] Sawa M, Masai H (2009). Drug design with Cdc7 kinase: a potential novel cancer therapy target. Drug Des Dev Ther.

[4] Leman AR, Noguchi E (2013). The replication fork: understanding the eukaryotic replication machinery and the challenges to genome duplication. Genes (Basel).

[5] Zou L, Elledge SJ (2003). Sensing DNA damage through ATRIP recognition of RPA-ssDNA complexes. Science.

[6] Ball HL, Myers JS, Cortez D (2005). ATRIP binding to replication protein A-single-stranded DNA promotes ATR–ATRIP localization but is dispensable for Chk1 phosphorylation. Mol Biol Cell.

[7] Kumagai A, Dunphy WG (2000). Claspin, a novel protein required for the activation of Chk1 during a DNA replication checkpoint response in Xenopus egg extracts. Mol Cell.

[8] Jeong SY, Kumagai A, Lee J, Dunphy WG (2003). Phosphorylated claspin interacts with a phosphate-binding site in the kinase domain of Chk1 during ATR-mediated activation. J Biol Chem.

[9] Kumagai A, Dunphy WG (2003). Repeated phosphopeptide motifs in Claspin mediate the regulated binding of Chk1. Nat Cell Biol.

[10] Lee J, Kumagai A, Dunphy WG (2003). Claspin, a Chk1-regulatory protein, monitors DNA replication on chromatin independently of RPA, ATR, and Rad17. Mol Cell.

[11] Petermann E, Caldecott KW (2006). Evidence that the ATR/Chk1 pathway maintains normal replication fork progression during unperturbed S phase. Cell Cycle.

[12] Liu S, Bekker-Jensen S, Mailand N, Lukas C, Bartek J, Lukas J (2006). Claspin operates downstream of TopBP1 to direct ATR signaling towards Chk1 activation. Mol Cell Biol.

[13] Zhao H, Piwnica-Worms H (2001). ATR-mediated checkpoint pathways regulate phosphorylation and activation of human Chk1. Mol Cell Biol.

[14] Lanz MC, Dibitetto D, Smolka MB (2019). DNA damage kinase signaling: checkpoint and repair at 30 years. EMBO J.

[15] Lecona E, Fernandez-Capetillo O (2018). Targeting ATR in cancer. Nat Rev Cancer.

[16] Tawfic S, Yu S, Wang H, Faust R, Davis A, Ahmed K (2001). Protein kinase CK2 signal in neoplasia. Histol Histopathol.

[17] Guerra B, Issinger OG (1999). Protein kinase CK2 and its role in cellular proliferation, development and pathology. Electrophoresis.

[18] Guerra BaIO-G (2013). CK2: a global regulator of cell survival. Protein kinase CK2.

[19] Wang H, Davis A, Yu S, Ahmed K (2001). Response of cancer cells to molecular interruption of the CK2 signal. Mol Cell Biochem.

[20] Ahmed K, Gerber DA, Cochet C (2002). Joining the cell survival squad: an emerging role for protein kinase CK2. Trends Cell Biol.

[21] Bibby AC, Litchfield DW (2005). The multiple personalities of the regulatory subunit of protein kinase CK2: CK2 dependent and CK2 independent roles reveal a secret identity for CK2beta. Int J Biol Sci.

[22] Schaefer S, Doktor TK, Frederiksen SB, Chea K, Hlavacova M, Bruun GH (2019). Down-regulation of CK2alpha correlates with decreased expression levels of DNA replication minichromosome maintenance protein complex (MCM) genes. Sci Rep.

[23] Lou DY, Dominguez I, Toselli P, Landesman-Bollag E, O'Brien C, Seldin DC (2008). The alpha catalytic subunit of protein kinase CK2 is required for mouse embryonic development. Mol Cell Biol.

[24] Xu X, Toselli PA, Russell LD, Seldin DC (1999). Globozoospermia in mice lacking the casein kinase II alpha' catalytic subunit. Nat Genet.

[25] Litchfield DW, Luscher B (1993). Casein kinase II in signal transduction and cell cycle regulation. Mol Cell Biochem.

[26] Pepperkok R, Lorenz P, Ansorge W, Pyerin W (1994). Casein kinase II is required for transition of G0/G1, early G1, and G1/S phases of the cell cycle. J Biol Chem.

[27] Guerra B, Issinger OG, Wang JY (2003). Modulation of human checkpoint kinase Chk1 by the regulatory beta-subunit of protein kinase CK2. Oncogene.

[28] Yde CW, Olsen BB, Meek D, Watanabe N, Guerra B (2008). The regulatory beta-subunit of protein kinase CK2 regulates cell-cycle progression at the onset of mitosis. Oncogene.

[29] Loizou JI, El-Khamisy SF, Zlatanou A, Moore DJ, Chan DW, Qin J (2004). The protein kinase CK2 facilitates repair of chromosomal DNA single-strand breaks. Cell.

[30] Melander F, Bekker-Jensen S, Falck J, Bartek J, Mailand N, Lukas J (2008). Phosphorylation of SDT repeats in the MDC1 N terminus triggers retention of NBS1 at the DNA damage-modified chromatin. J Cell Biol.

[31] Olsen BB, Issinger OG, Guerra B (2010). Regulation of DNA-dependent protein kinase by protein kinase CK2 in human glioblastoma cells. Oncogene.

[32] Olsen BB, Wang SY, Svenstrup TH, Chen BP, Guerra B (2012). Protein kinase CK2 localizes to sites of DNA double-strand break regulating the cellular response to DNA damage. BMC Mol Biol.

[33] Guerra B, Iwabuchi K, Issinger OG (2014). Protein kinase CK2 is required for the recruitment of 53BP1 to sites of DNA double-strand break induced by radiomimetic drugs. Cancer Lett.

[34] Guerra B, Dembic M, Siddiqui MA, Dominguez I, Ceppi P, Andresen BS (2020). Down-regulation of CK2alpha leads to up-regulation of the cyclin-dependent kinase inhibitor p27(KIP1) in conditions unfavorable for the growth of myoblast cells. Cell Physiol Biochem.

[35] Peschiaroli A, Dorrello NV, Guardavaccaro D, Venere M, Halazonetis T, Sherman NE (2006). SCFbetaTrCP-mediated degradation of claspin regulates recovery from the DNA replication checkpoint response. Mol Cell.

[36] Mendez J, Stillman B (2000). Chromatin association of human origin recognition complex, cdc6, and minichromosome maintenance proteins during the cell cycle: assembly of prereplication complexes in late mitosis. Mol Cell Biol.

[37] Guerra B, Siemer S, Boldyreff B, Issinger OG (1999). Protein kinase CK2: evidence for a protein kinase CK2beta subunit fraction, devoid of the catalytic CK2alpha subunit, in mouse brain and testicles. FEBS Lett.

[38] Olsen BB, Guerra B (2008). Ability of CK2beta to selectively regulate cellular protein kinases. Mol Cell Biochem.

[39] Bushnell B (2014) BBMap: a fast, accurate, splice-aware aligner. United States. http://www.osti.gov/servlets/purl/1241166

[40] Patro R, Duggal G, Love MI, Irizarry RA, Kingsford C (2017). Salmon provides fast and bias-aware quantification of transcript expression. Nat Methods.

[41] Soneson C, Love MI, Robinson MD (2015). Differential analyses for RNA-seq: transcript-level estimates improve gene-level inferences. F1000Res.

[42] Love MI, Huber W, Anders S (2014). Moderated estimation of fold change and dispersion for RNA-seq data with DESeq2. Genome Biol.

[43] Zhu A, Ibrahim JG, Love MI (2019). Heavy-tailed prior distributions for sequence count data: removing the noise and preserving large differences. Bioinformatics.

[44] Wehrens R, Buydens LM (2007). Self- and super-organizing maps in R: the Kohonen package. J Stat Softw.

[45] Wehrens R, Kruisselbrink J (2018). Flexible self-organizing maps in Kohonen 3.0. J Stat Softw.

[46] Yu G, Wang LG, Han Y, He QY (2012). clusterProfiler: an R package for comparing biological themes among gene clusters. OMICS.

[47] Luo W, Brouwer C (2013). Pathview: an R/Bioconductor package for pathway-based data integration and visualization. Bioinformatics.

[48] Somyajit K, Gupta R, Sedlackova H, Neelsen KJ, Ochs F, Rask MB (2017). Redox-sensitive alteration of replisome architecture safeguards genome integrity. Science.

[49] Vesela E, Chroma K, Turi Z, Mistrik M (2017). Common chemical inductors of replication stress: focus on cell-based studies. Biomolecules.

[50] Ward IM, Minn K, Chen J (2004). UV-induced ataxia-telangiectasia-mutated and Rad3-related (ATR) activation requires replication stress. J Biol Chem.

[51] Kelly TJ, Brown GW (2000). Regulation of chromosome replication. Annu Rev Biochem.

[52] Walter J, Newport J (2000). Initiation of eukaryotic DNA replication: origin unwinding and sequential chromatin association of Cdc45, RPA, and DNA polymerase alpha. Mol Cell.

[53] Cortez D, Glick G, Elledge SJ (2004). Minichromosome maintenance proteins are direct targets of the ATM and ATR checkpoint kinases. Proc Natl Acad Sci USA.

[54] Ibarra A, Schwob E, Mendez J (2008). Excess MCM proteins protect human cells from replicative stress by licensing backup origins of replication. Proc Natl Acad Sci USA.

[55] Kruse JP, Gu W (2009). Modes of p53 regulation. Cell.

[56] Liu P, Barkley LR, Day T, Bi X, Slater DM, Alexandrow MG (2006). The Chk1-mediated S-phase checkpoint targets initiation factor Cdc45 via a Cdc25A/Cdk2-independent mechanism. J Biol Chem.

[57] Bruck I, Kaplan DL (2013). Cdc45 protein-single-stranded DNA interaction is important for stalling the helicase during replication stress. J Biol Chem.

[58] Broderick R, Rainey MD, Santocanale C, Nasheuer HP (2013). Cell cycle-dependent formation of Cdc45-Claspin complexes in human cells is compromized by UV-mediated DNA damage. FEBS J.

[59] Takaya J, Kusunoki S, Ishimi Y (2013). Protein interaction and cellular localization of human CDC45. J Biochem.

[60] Bacevic K, Lossaint G, Achour TN, Georget V, Fisher D, Dulic V (2017). Cdk2 strengthens the intra-S checkpoint and counteracts cell cycle exit induced by DNA damage. Sci Rep.

[61] Bauerschmidt C, Pollok S, Kremmer E, Nasheuer HP, Grosse F (2007). Interactions of human Cdc45 with the Mcm2-7 complex, the GINS complex, and DNA polymerases delta and epsilon during S phase. Genes Cells.

[62] Tsao CC, Geisen C, Abraham RT (2004). Interaction between human MCM7 and Rad17 proteins is required for replication checkpoint signaling. EMBO J.

[63] Ball HL, Cortez D (2005). ATRIP oligomerization is required for ATR-dependent checkpoint signaling. J Biol Chem.

[64] Bomgarden RD, Yean D, Yee MC, Cimprich KA (2004). A novel protein activity mediates DNA binding of an ATR–ATRIP complex. J Biol Chem.

[65] Cortez D, Guntuku S, Qin J, Elledge SJ (2001). ATR and ATRIP: partners in checkpoint signaling. Science.

[66] Kumagai A, Kim SM, Dunphy WG (2004). Claspin and the activated form of ATR–ATRIP collaborate in the activation of Chk1. J Biol Chem.

[67] Smits VAJ, Cabrera E, Freire R, Gillespie DA (2019). Claspin-checkpoint adaptor and DNA replication factor. FEBS J.

[68] Chini CC, Chen J (2003). Human claspin is required for replication checkpoint control. J Biol Chem.

[69] Lin SY, Li K, Stewart GS, Elledge SJ (2004). Human Claspin works with BRCA1 to both positively and negatively regulate cell proliferation. Proc Natl Acad Sci U S A.

[70] Stracker TH, Usui T, Petrini JH (2009). Taking the time to make important decisions: the checkpoint effector kinases Chk1 and Chk2 and the DNA damage response. DNA Repair (Amst).

[71] Smits VA (2006). Spreading the signal: dissociation of Chk1 from chromatin. Cell Cycle.

[72] Smits VA, Reaper PM, Jackson SP (2006). Rapid PIKK-dependent release of Chk1 from chromatin promotes the DNA-damage checkpoint response. Curr Biol.

[73] Tenca P, Brotherton D, Montagnoli A, Rainoldi S, Albanese C, Santocanale C (2007). Cdc7 is an active kinase in human cancer cells undergoing replication stress. J Biol Chem.

[74] Rainey MD, Harhen B, Wang GN, Murphy PV, Santocanale C (2013). Cdc7-dependent and -independent phosphorylation of Claspin in the induction of the DNA replication checkpoint. Cell Cycle.

[75] Huh J, Piwnica-Worms H (2013). CRL4(CDT2) targets CHK1 for PCNA-independent destruction. Mol Cell Biol.

[76] Schwarzerova K, Bellinvia E, Martinek J, Sikorova L, Dostal V, Libusova L (2019). Tubulin is actively exported from the nucleus through the Exportin1/CRM1 pathway. Sci Rep.

[77] Ward IM, Chen J (2001). Histone H2AX is phosphorylated in an ATR-dependent manner in response to replicational stress. J Biol Chem.

[78] Gagou ME, Zuazua-Villar P, Meuth M (2010). Enhanced H2AX phosphorylation, DNA replication fork arrest, and cell death in the absence of Chk1. Mol Biol Cell.

[79] Syljuasen RG, Sorensen CS, Hansen LT, Fugger K, Lundin C, Johansson F (2005). Inhibition of human Chk1 causes increased initiation of DNA replication, phosphorylation of ATR targets, and DNA breakage. Mol Cell Biol.

[80] Kumagai A, Lee J, Yoo HY, Dunphy WG (2006). TopBP1 activates the ATR–ATRIP complex. Cell.

[81] Feng H, Lu J, Song X, Thongkum A, Zhang F, Lou L (2020). CK2 kinase-mediated PHF8 phosphorylation controls TopBP1 stability to regulate DNA replication. Nucleic Acids Res.

[82] Hao J, de Renty C, Li Y, Xiao H, Kemp MG, Han Z (2015). And-1 coordinates with Claspin for efficient Chk1 activation in response to replication stress. EMBO J.

[83] Chini CC, Wood J, Chen J (2006). Chk1 is required to maintain claspin stability. Oncogene.

[84] Buchsbaum S, Morris C, Bochard V, Jalinot P (2007). Human INT6 interacts with MCM7 and regulates its stability during S phase of the cell cycle. Oncogene.

[85] Chuang CH, Yang D, Bai G, Freeland A, Pruitt SC, Schimenti JC (2012). Post-transcriptional homeostasis and regulation of MCM2-7 in mammalian cells. Nucleic Acids Res.

[86] Montagnoli A, Valsasina B, Brotherton D, Troiani S, Rainoldi S, Tenca P (2006). Identification of Mcm2 phosphorylation sites by S-phase-regulating kinases. J Biol Chem.

[87] Pollok S, Grosse F (2007). Cdc45 degradation during differentiation and apoptosis. Biochem Biophys Res Commun.

[88] Pollok S, Bauerschmidt C, Sanger J, Nasheuer HP, Grosse F (2007). Human Cdc45 is a proliferation-associated antigen. FEBS J.

[89] Arata Y, Fujita M, Ohtani K, Kijima S, Kato JY (2000). Cdk2-dependent and -independent pathways in E2F-mediated S phase induction. J Biol Chem.

[90] Leone G, DeGregori J, Yan Z, Jakoi L, Ishida S, Williams RS (1998). E2F3 activity is regulated during the cell cycle and is required for the induction of S phase. Genes Dev.

[91] Ohtani K, Iwanaga R, Nakamura M, Ikeda M, Yabuta N, Tsuruga H (1999). Cell growth-regulated expression of mammalian MCM5 and MCM6 genes mediated by the transcription factor E2F. Oncogene.

[92] Burkhart DL, Wirt SE, Zmoos AF, Kareta MS, Sage J (2010). Tandem E2F binding sites in the promoter of the p107 cell cycle regulator control p107 expression and its cellular functions. PLoS Genet.

[93] Woo RA, Poon RY (2003). Cyclin-dependent kinases and S phase control in mammalian cells. Cell Cycle.

[94] Walter JC (2000). Evidence for sequential action of cdc7 and cdk2 protein kinases during initiation of DNA replication in Xenopus egg extracts. J Biol Chem.

[95] Li X, Qian X, Jiang H, Xia Y, Zheng Y, Li J (2018). Nuclear PGK1 alleviates ADP-dependent inhibition of CDC7 to promote DNA replication. Mol Cell.

[96] Halicka HD, Huang X, Traganos F, King MA, Dai W, Darzynkiewicz Z (2005). Histone H2AX phosphorylation after cell irradiation with UV-B: relationship to cell cycle phase and induction of apoptosis. Cell Cycle.

