# Supplementary figures and images for: Essential role of CK2α for the interaction and stability of replication fork factors during DNA synthesis and activation of the S-phase checkpoint
Source: Cell Mol Life Sci. 2022 Jun 4;79(6):339. doi: 10.1007/s00018-022-04374-3 (PMC9166893; doi:10.1007/s00018-022-04374-3)

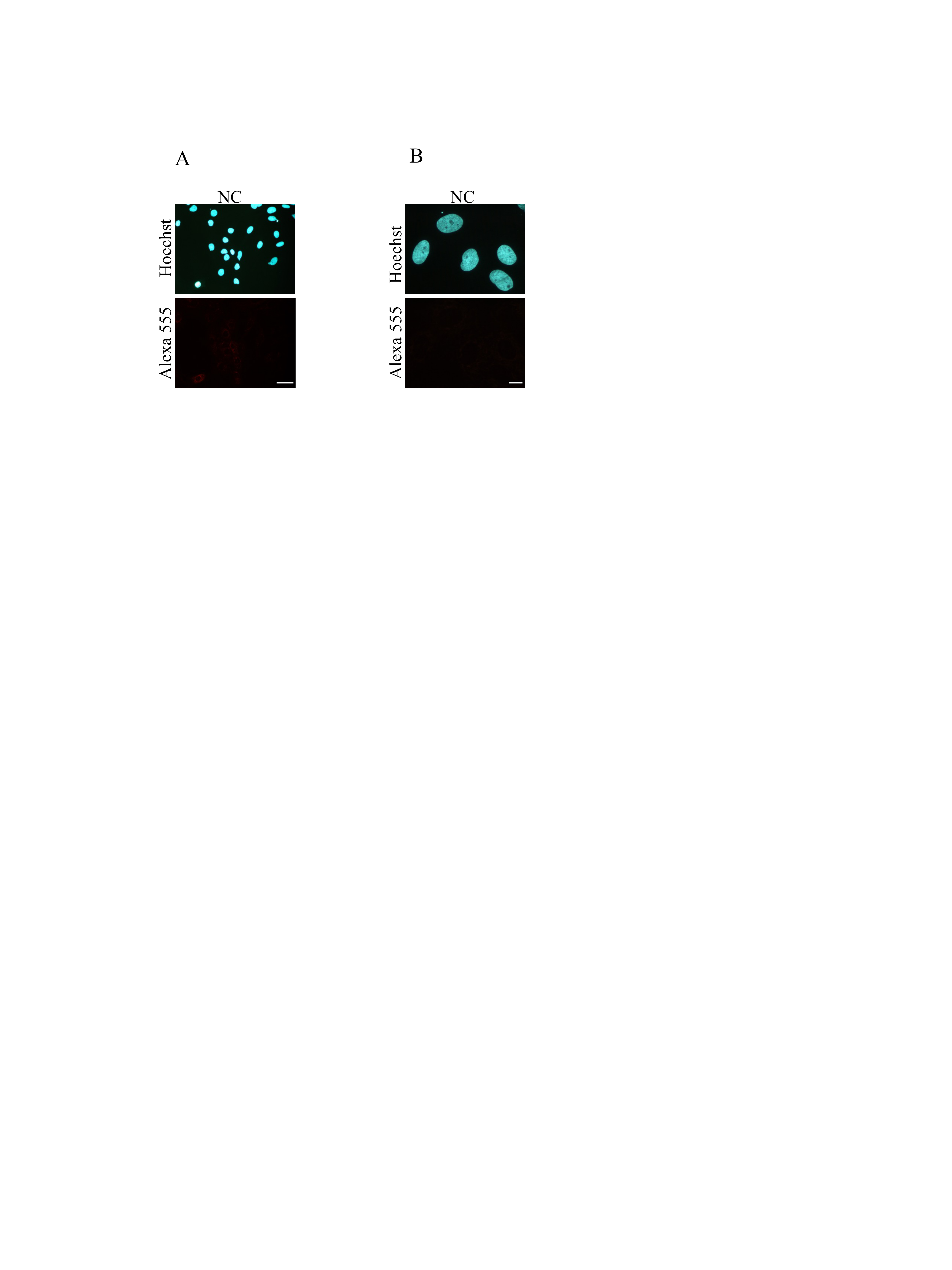

Supplement: Supplementary file 1 — Supplementary file1 Suppl. Fig S1. Expression pattern of phosphorylated CHK1 and H2AX in cells - negative controls. A Negative control (NC) for phospho-CHK1 (S345) staining of myoblasts cells. Images were taken at 20x magnification. Scale bar represents 50 μm. B Negative control for phospho-H2AX (S139) staining of myoblasts cells. Images were taken at 40x magnification. Scale bar represents 20 μm (TIFF 851 KB) [file 18_2022_4374_MOESM1_ESM.tiff]

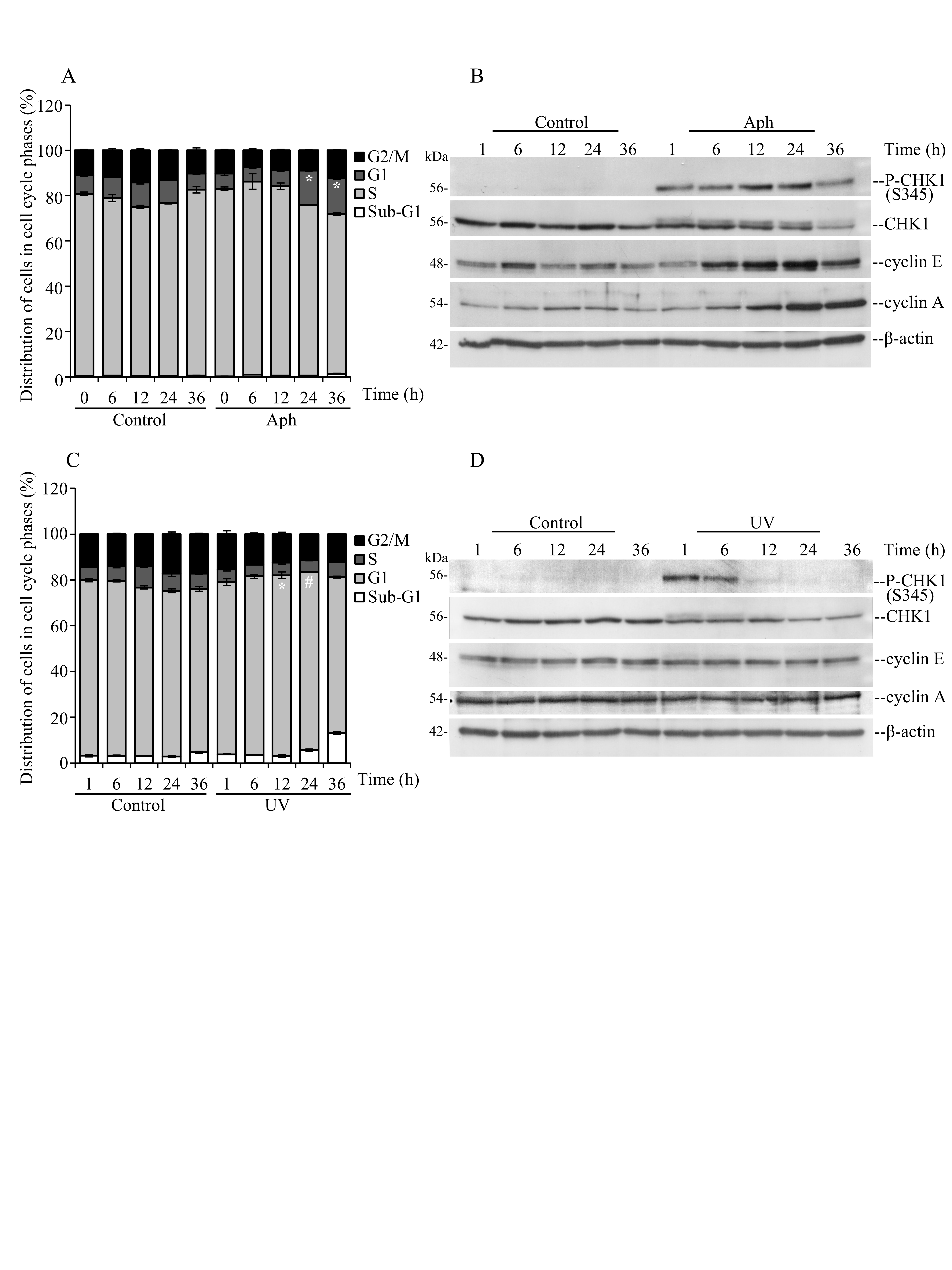

Supplement: Supplementary file 2 — Supplementary file2 Suppl. Fig S2. Response of myoblasts to treatment with aphidicolin or exposure to UV irradiation. A, C Cells were treated with 5 μM aphidicolin (Aph) to inhibit DNA polymerase (A) or exposed to 60 J/m2 UV irradiation (C) to induce DNA replication stress [49]. Cells were harvested at different time points and analysed by Flow cytometry after staining the DNA with propidium iodide. *P < 0.005 with respect to control cells at 24 h, and 36 h, respectively (A), *P = 0.05 with respect to control cells at 12 h and #P < 0.005 with respect to control cells at 24 h (C). B, D Western blot analysis of whole lysates from cells treated essentially as indicated above, was carried out employing antibodies against proteins indicated in the figure. β-actin detection served as loading control. (TIFF 3334 KB) [file 18_2022_4374_MOESM2_ESM.tiff]

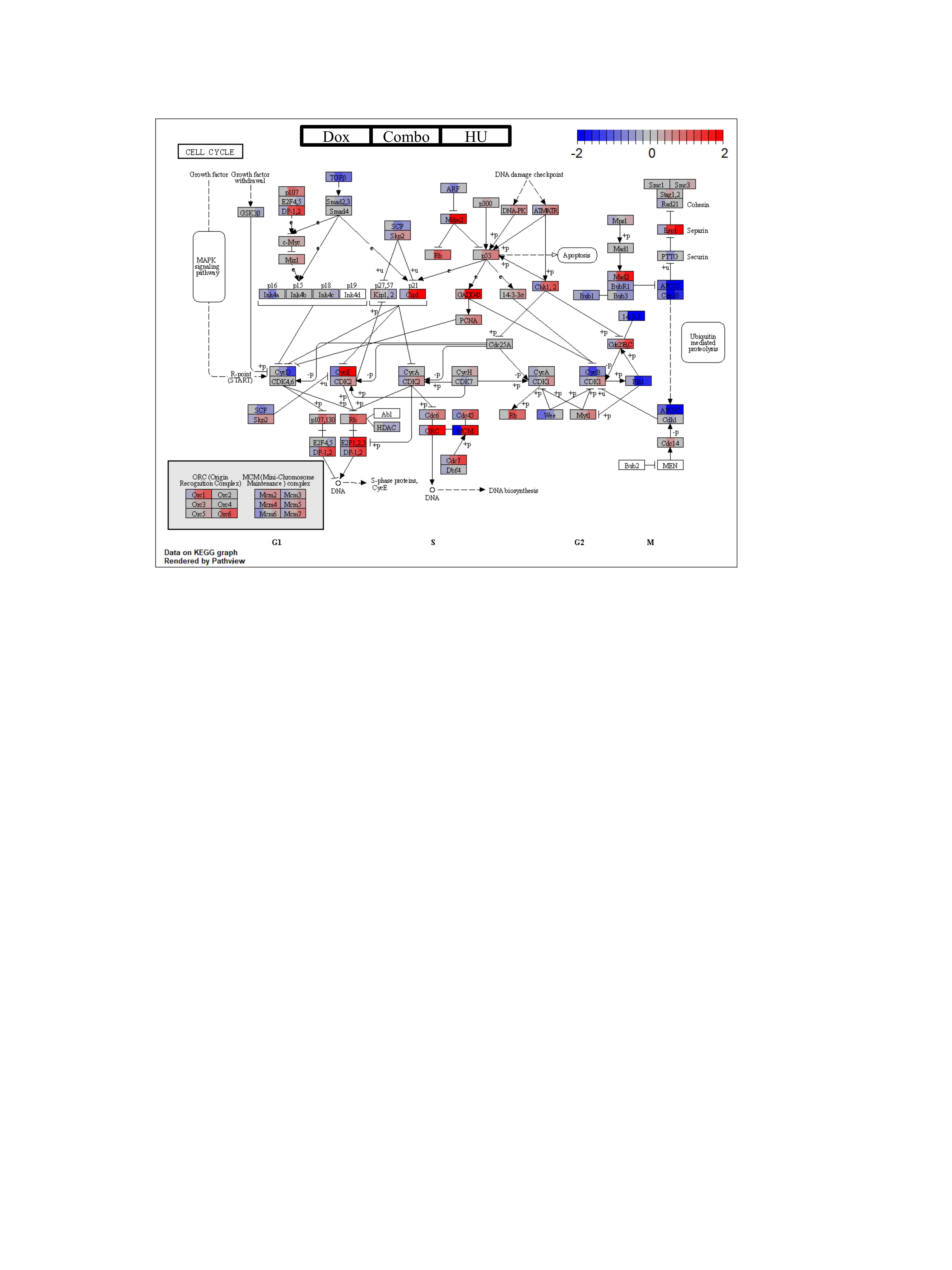

Supplement: Supplementary file 3 — Supplementary file3 Suppl. Fig S3. Gene expression changes within the cell cycle pathway following treatment of H9c2-CK2α-44 with Dox, HU, or a combination. Following treatment of cells with either 1 μg/ml Dox for 72 h, 3 mM HU for 24 h or a combination, RNA-sequencing was used to obtain log2 fold-change estimates of gene expression changes relative to untreated controls. The changes in gene expression are indicated in colour, with bright red indicating a positive log2 fold-change of at least 2, and bright blue a negative log2 fold-change of at least 2. The three conditions are displayed such that for each gene or gene-group, the first colour shows the change in gene expression following Dox treatment alone, the second colour the combination of Dox and HU, and the last colour the effect on gene expression following treatment with HU alone.(TIFF 1247 KB) [file 18_2022_4374_MOESM3_ESM.tiff]

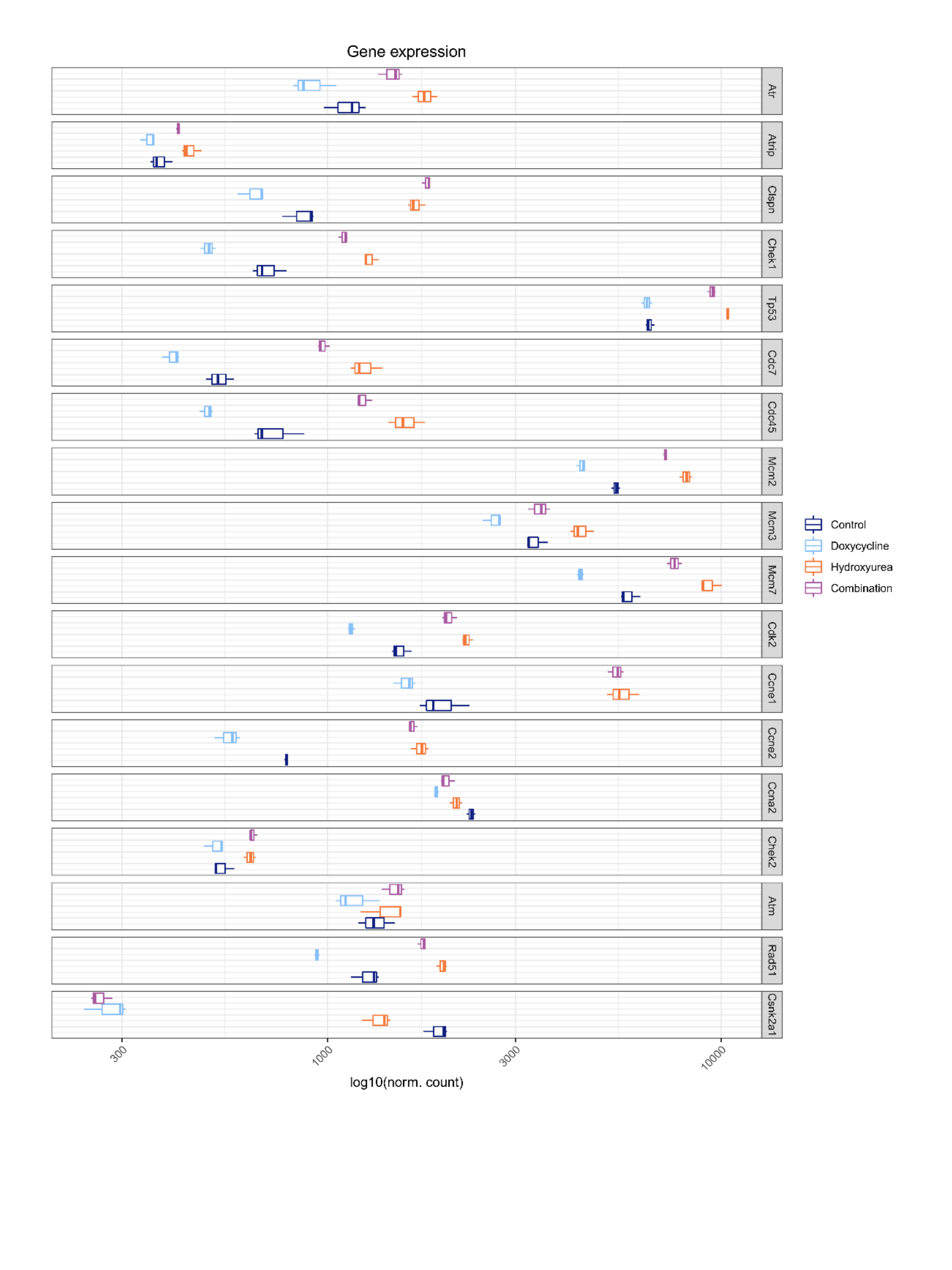

Supplement: Supplementary file 4 — Supplementary file4 Suppl. Fig S4. Gene expression changes relative to proteins previously examined by Western blot. Cells were treated as indicated in Suppl. Fig S3. RNA-sequencing was used to obtain normalized expression estimates of genes coding for proteins examined by Western blot. The four different conditions are indicated by colour as indicated in the figure. Gene expression estimates were log10 transformed to plot highly differing gene expression estimates within the same plot.(TIFF 1344 KB) [file 18_2022_4374_MOESM4_ESM.tiff]

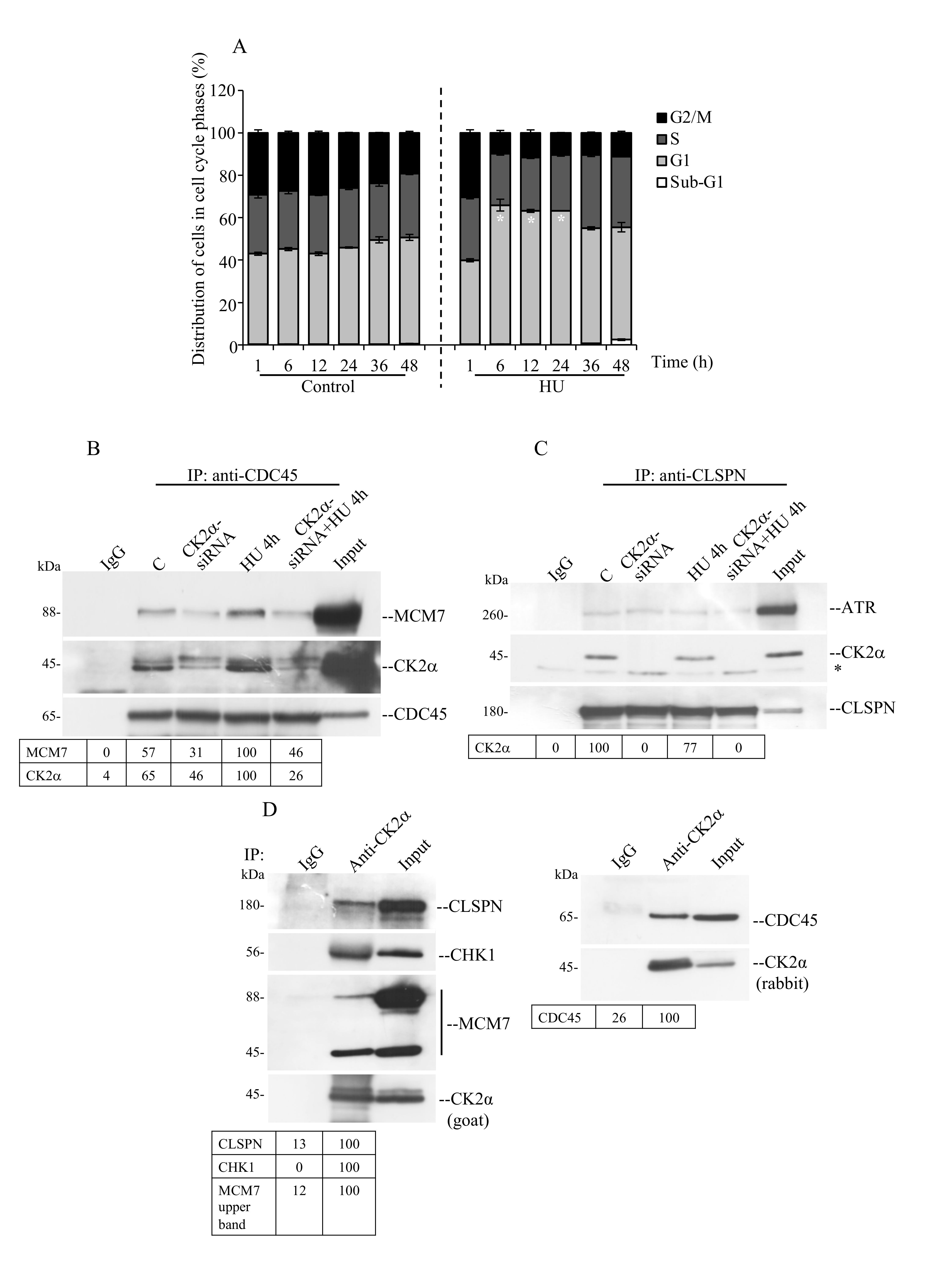

Supplement: Supplementary file 5 — Supplementary file5 Suppl. Fig S5. Analysis of complex formations in U2OS cells following down-regulation of CK2α and DNA replication stress induction. A U2OS cells were treated with 3 mM HU and harvested at the indicated time points. *P < 0.005 with respect to control cells at 6 h, 12 h, and 24 h, respectively. Experiments were subsequently carried out as described in Fig 1A. B, C Cells were transfected for 56 h with CK2α-siRNA to induce down-regulation of the protein kinase. It followed incubation with 3 mM HU for additional 4 h before harvesting. Whole cell lysates were subsequently subjected to immunoprecipitation assays essentially as described in Fig 5 with anti-CDC45 (B) and anti-CLSPN antibodies (C), respectively. D Whole cell lysate from U2OS cells was employed in immunoprecipitation studies as described in Fig 5H. The identification of the co-precipitated proteins was carried out by Western blot employing antibodies indicated in the figure. In the analysis of the immunoblots, we noticed that the detection of MCM7 resulted in two band signals corresponding to proteins with distinct molecular weights suggesting co-immunoprecipitation of full length and a shorter form of MCM7. This is plausible as alternatively spliced transcript variants encoding distinct isoforms of MCM7 have been reported (http://atlasgeneticsoncology.org/Genes/GC_MCM7.html). (TIFF 1939 KB) [file 18_2022_4374_MOESM5_ESM.tiff]

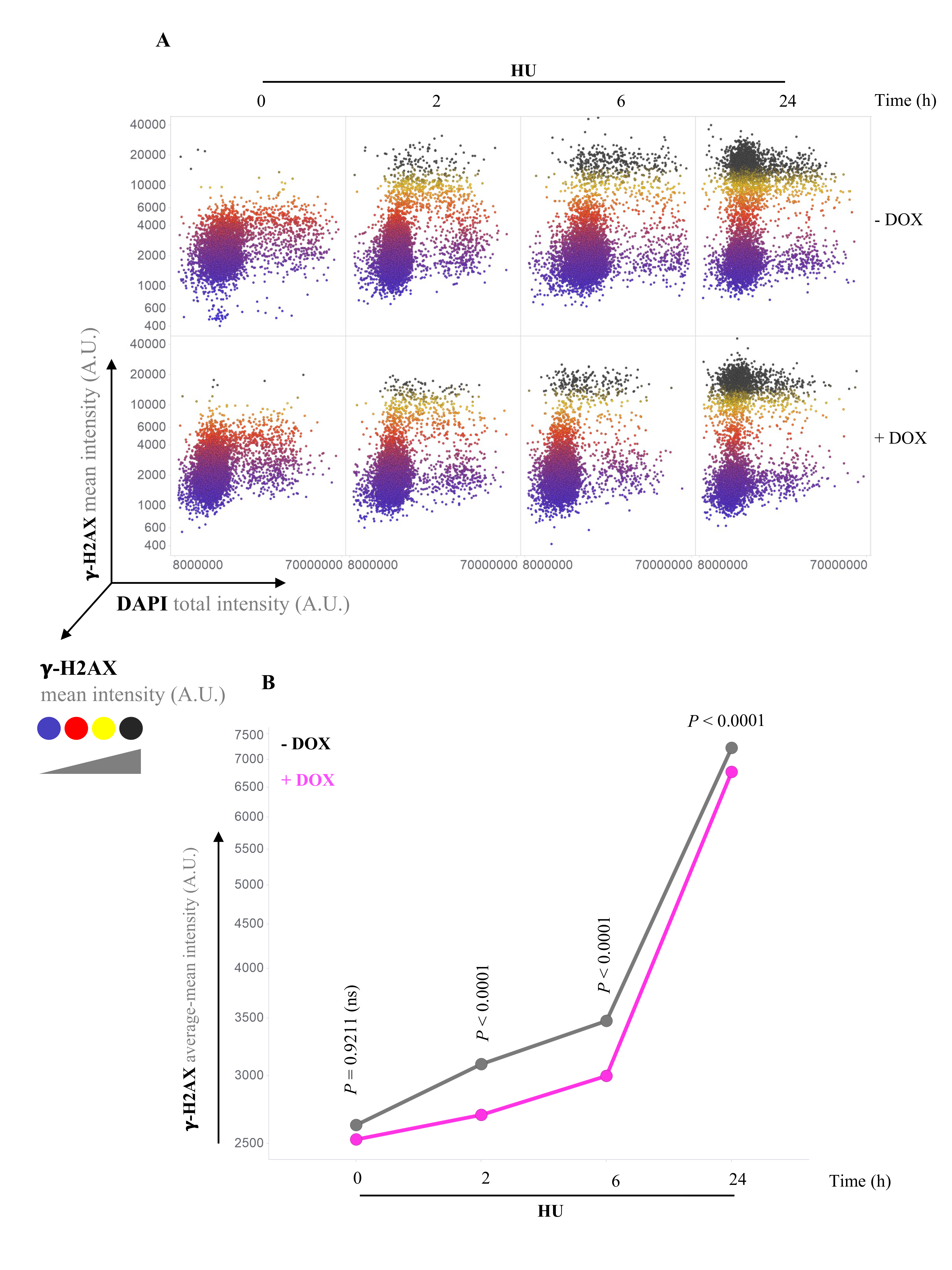

Supplement: Supplementary file 6 — Supplementary file6 Suppl. Fig S6. Analysis of cells by quantitative image-based cytometry (QIBC) reveals decreased levels of phosphorylation of H2AX in cells with down-regulation of CK2α and exposed to HU. A QIBC analysis of cells exposed to 3 mM HU for the indicated times and immunostained for γ-H2AX. Nuclear DNA was counterstained by 4′,6-diamidino-2-phenylindole (DAPI), n >4,000 cells for each condition. The colour gradient indicates the mean nuclear γ-H2AX intensity. A.U., arbitrary units. B Quantification of γ-H2AX signal intensity from the experiment performed in A. The data points represent average values. n > 4,000 cells. Statistical analysis were done with GraphPad Prism (GraphPad Software version 9) using one-way ANOVA. (TIFF 8428 KB) [file 18_2022_4374_MOESM6_ESM.tiff]
